# Impact of high wind speed on blooming plants-honeybees-honey production model 

**DOI:** 10.12688/f1000research.172134.1

**Published:** 2025-12-26

**Authors:** Shireen Jawad, Zainab Hayder Abid AL-Aali

**Affiliations:** 1 Mathematics, University of Baghdad, Baghdad, Baghdad Governorate, Iraq

**Keywords:** Wind speed, blooming plants-honeybees model, mutualistic relationship, Beddington-DeAngelis functional response, dynamical systems, stability analysis, bifurcation.

## Abstract

**Background:**

Local ecosystems and global agriculture are contingent upon the mutualistic relationship between pollinators and floral plants. In symbiosis, pollinators increase agricultural production by improving plant cross-pollination, genetic variety, crop quality, and yield. The potential impact on plant reproduction is particularly alarming due to the decline of pollinating insects. Habitat loss, diseases, climate change, pesticides, and predation have all contributed to the decline of pollinator species. High-speed wind is a significant factor that impacts the mutualistic relationship between plants and pollinators.

**Methods:**

Studying the dynamics of interactions between blooming plants and honeybee populations is crucial for addressing honeybee decline and ensuring sustainable ecosystems. This work employs mathematical modeling to analyze the dynamics of a blooming plant, honeybee population, and honey production symbiosis, with a special emphasis on the effect of high-speed wind flow.

**Results:**

The stability of various ecological equilibria has been investigated using dynamical system theory. Bifurcation phenomena, such as transcritical and Hopf bifurcations, have been discovered using bifurcation theory. Furthermore, the numerical results show that high wind flow can cause the extinction of the honeybee population and honey production.

**Conclusions:**

Due to the rapid depletion of flowering plants and the high rate of wind speed, the populations of honeybees and blossoming plants are at risk of becoming unsustainable. However, the combination of reduced wind flow and increased symbiotic strengths can bolster the stability and sustainability of blooming plant-honeybee-honey production ecosystems. These findings inform conservation policies targeted toward protecting honeybees and increasing biodiversity.

## Introduction

The mutualistic relationship between blooming plants and honeybee populations is a crucial ecological interaction for the sustainability of both local ecosystems and global agricultural systems. ^1^ Animal pollinators, including honeybees, provide pollination services through symbiosis, which is essential for the successful reproduction of approximately 300,000 plant species worldwide. In symbiosis, pollinators are vital for promoting plant cross-pollination, genetic diversity, and crop quality and yield, substantially contributing to agricultural productivity. ^2^ Consequently, this is a critical research area in conservation biology and ecology. A significant number of studies have been undertaken regarding the symbiotic relationships between plants and pollinator systems. ^3^ Hadani ^4^ suggested that the symbiosis can be characterized by the Beddington-DeAngelis response function, which incorporates competition for resource exploitation among pollinators and the obligatory relationship between the plant and the pollinator. This relationship has been observed to maintain a steady state, provided that the initial population level is sufficiently substantial. ^5^ Biswas et al. examined a plant–pollinator model to investigate the impact of predation on pollinator species. They conclude that the pollinator is at risk of extinction if the predation rate cannot be controlled. Moreover, this hypothesis has prompted the advancement of extensive studies examining the impacts of nectar theft and ants on the plant-pollinator system. ^6^ The reduction of biodiversity is a widespread issue, although the decrease of pollinating insects is especially alarming due to its possible effects on plant reproduction. ^7^ A recent report on the global reduction of honeybee and bumblebee populations has highlighted pollination’s ecological and economic significance. ^8^ The reduction of pollinator species can be ascribed to various ecological and environmental reasons, including habitat loss, illnesses, climate change, pesticides, and predation. ^9– 12^ Invasive predators can significantly affect pollinators by diminishing their quantity, altering plant reproductive success, and undermining the plant-pollinator relationship. ^13^

One factor that negatively affects the mutualism in plant-pollinator systems is high-speed wind. ^5, 14– 17^ High winds disrupt flower fragrance messages, reducing honeybee attraction. Strong winds quickly distribute flowers’ aromatic chemicals in different directions, reducing their “scent signal”. As aroma dispersion reduces, honeybees’ ability to discover food sources decreases, resulting in fewer trips to flowers. ^18^ In addition, flying in severe winds demands more energy for balance and advancement. Honeybees may stay in the hive or fly less to preserve energy. Pollination opportunities decrease as fewer flowers are visited. Strong winds can break flowers’ petals or open their parts abnormally, decreasing honeybees’ access to their reproductive organs. This temporal shift may diminish pollination when flowers are most pollinating. High winds can cause honeybees to crash, fall, or be blown off course, destroying the colony. Lost workers or persistent stress can harm the colony and its capacity to provide enough workers for visits. ^19^

In this study, we discuss the effect of high wind speed on a three-dimensional blooming plant–honeybee–honey production mathematical model ( phn model) that takes into account saturated mutualism between blooming plants and honeybees by the Beddington–DeAngelis response function. The primary focus is to investigate the dynamics of the plant–pollinator system while considering the impacts of wind speed on the mutualism between blooming plants and honeybees. The study aims to understand the interactions between mutualism and wind speed, and how these interactions affect the overall ecological balance and sustainability of the blooming plant–honeybee–honey production system.

## Methods

### Assumptions of the model

In this section, a phn model is formulated to describe the interaction among blooming plants p(t) , honey honeybees h(t) and the production of honey n(t) at time t . Then the blooming plant, the honey honeybees, and the production of honey system can be depicted by

$$\begin{array}{l} \frac{\mathrm{dp}}{\mathrm{dt}}=r_{1}p\left(1-\frac{p}{k_{1}}\right)+\frac{\alpha_{1}\mathrm{hp}}{1+w+\left(\mathrm{ah}+\mathrm{bp}\right)}-\gamma_{1}p,\\ \frac{\mathrm{dh}}{\mathrm{dt}}=r_{2}h\left(1-\frac{h}{k_{2}}\right)+\frac{\alpha_{2}\mathrm{hp}}{1+w+\left(\mathrm{ah}+\mathrm{bp}\right)}-\gamma_{2}h,\\ \frac{\mathrm{dn}}{\mathrm{dt}}=\frac{\alpha_{3}h}{1+w+\mathrm{ch}}-\mathrm{\beta nh}-\gamma_{3}n. \end{array}$$ (1)

1. The blooming plants are assumed to grow in the absence of honeybees at the intrinsic growth rate $r_{1}$ , depletion rate $\gamma_{1}$ and carrying capacity $k_{1}$ . Since blooming plants provide nutrients for honeybees, honeybees offer pollination services to blooming plants; hence, their relationship is mutualistic. Beddington-DeAngelis functional response $\left(\frac{\alpha_{1}\mathrm{hp}}{1+w+\left(\mathrm{ah}+\mathrm{bp}\right)}\right)$ can be used to express the mutualistic relationship, where $\alpha_{1}$ denotes the positive effect of honeybees (a kind of pollinator) on plants, a refers to the undepleted equilibrium rate for the blooming plant–honeybee interaction, which incorporates travel and unloading durations at a central location along with individual-level blooming plant–honeybee interactions, and b indicates the intensity of competition among honeybees for floral resources.
2. High winds break plants’ stems and branches, causing flowers to collapse and damage their petals. This damage reduces the flowers’ attractiveness to pollinating insects. Also, wind speeds reduce the honeybees’ ability to search in a windy environment. Let $\vartheta \left(w\right)=\frac{1}{1+w}$ be the efficiency of wind, which satisfied the following
  •
ϑ(0)=1 means in the absence of wind, the mutualistic interaction between blooming plants and honeybees remains as before, i.e., $\left(\frac{\alpha_{1}\mathrm{hp}}{1+\left(\mathrm{ah}+\mathrm{bp}\right)}\right)$ .
  •
ϑ(w)>1 means the efficiency of mutualistic interaction decreases in a high, windy environment.
3. The honeybee population are expected to grow from external resources at the intrinsic growth rate $r_{2}$ , depletion rate $\gamma_{2}$ and carrying capacity $k_{2}$ . $\left(\frac{\alpha_{2}\mathrm{hp}}{1+w+\left(\mathrm{ah}+\mathrm{bp}\right)}\right)$ represents the nutrients that blooming plants provide to honeybees, where $\alpha_{2}$ stands for the corresponding value of honeybee nutrients from blooming plants.
4. The term $\left(\frac{\alpha_{3}h}{1+w+\mathrm{ch}}\right)$ represents the honey production in the colonies where $\alpha_{3}$ is the rate of production that is contingent upon the quantity of honeybees, and c is the half-saturation rate. Wind speed negatively affects the amount of honey produced since it diminishes the honeybees’ capacity to search for nutrition.
5. During winter or drought, honeybees rely on their stored honey to survive. This represents a natural consumption process but diminishes the quantity available for harvest. Therefore, β signifies the rate at which honeybees consume honey to survive.
6. The amount of honey produced decreases due to many factors, such as absorbing moisture from the atmosphere. In humid environments, honey absorbs moisture, which reduces its concentration and makes it susceptible to fermentation and spoilage. High temperatures cause the honey content to evaporate. Therefore, $\gamma_{3}$ denotes the rate of natural causes of honey loss.

**Figure 1.**
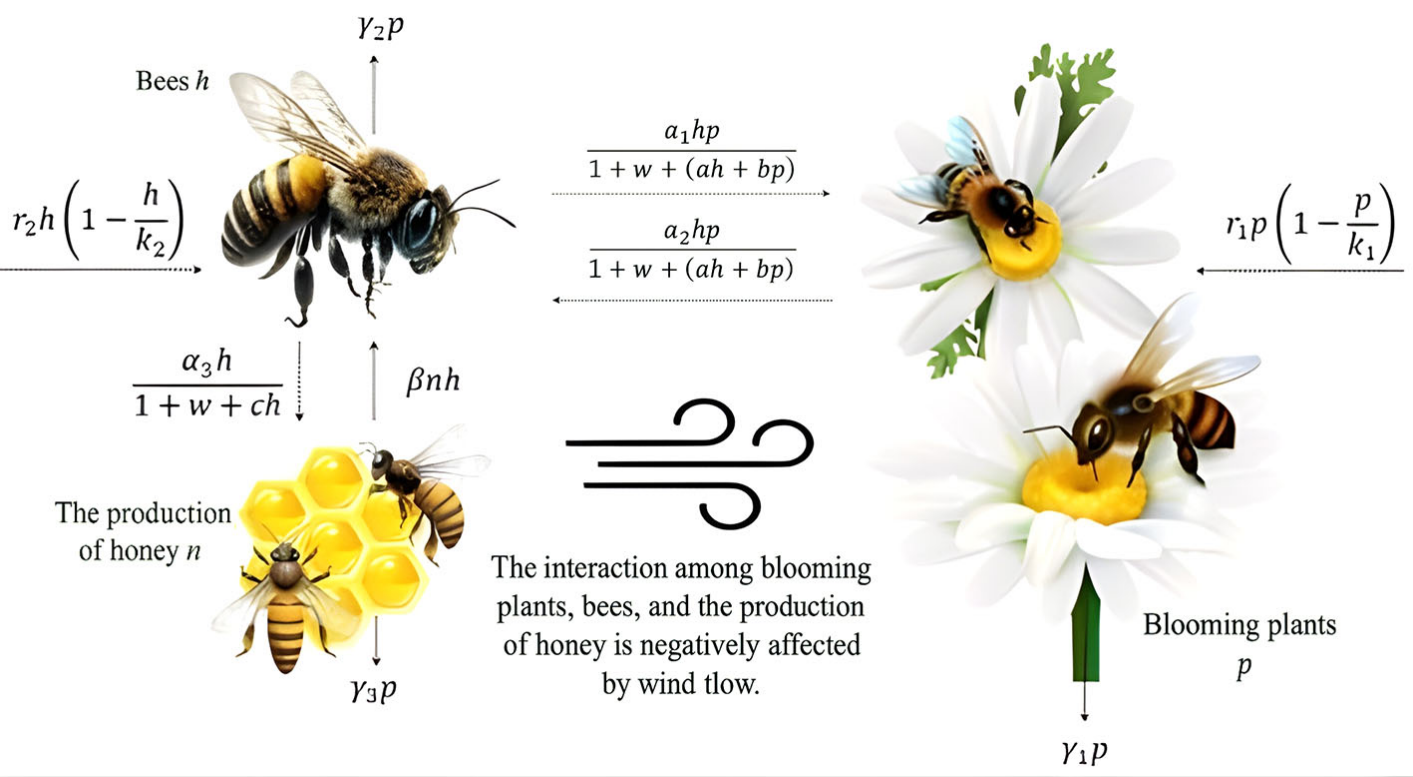
Flowchart of the phn model.

Further, the schematic sketch of phn system is illustrated in Figure 1.

### Model analysis

Before analyzing our model, it is pertinent to invoke the following lemmas, the demonstration of which is available in Refs. 20- 22.

Lemma 1. If H,ℊ>0 and $\dot{H}⩾\left(⩽\right)H\left(H-ℊH^{\alpha }\right)$ , where a is a positive constant, when t⩾0 and H(0)>0 , then

$$H\left(t\right)⩾\left(⩽\right){\left(\frac{H}{ℊ}\right)}^{1/\alpha }{\left[1+\left(\frac{HH^{-a}\left(0\right)}{ℊ}-1\right)e^{-H\mathrm{\alpha t}}\right]}^{-1/\alpha }$$

Lemma 2. (Comparison lemma) Suppose that H,ℊ>0, with Ρ(0)>0 . Then for $\frac{\mathrm{dΡ}}{\mathrm{dt}}\leq Ρ\left(t\right)\left[H-ℊ\;Ρ\left(t\right)\right]$ , then $\underset{t\to \infty }{\text{lim}}\text{sup}P\left(t\right)\leq \frac{H}{ℊ}$ , and if $\frac{dΡ}{\mathrm{dt}}\geq Ρ\left(t\right)\left[H-ℊ\;Ρ\left(t\right)\right]$ , then $\underset{t\to \infty }{\text{lim}}\text{inf}P\left(t\right)\geq \frac{H}{ℊ}$ .

Uniqueness.

Since the right side of the phn model $\;C^{1}\;\left(R_{+}^{3}\right)$ , so they satisfy the Lipschitz condition. Therefore, the solution to phn model that starts in $R_{+}^{3}$ exists and is unique.

### Positivity

Theorem 1. The solution (p(t),h(t),n(t)) of phn system with the initial condition $\left(p_{0},h_{0},n_{0}\right)$ is positive.

Proof. According to the blooming plants and honeybees’ equations of phn system, we get

$$p\left(t\right)=p_{0}e^{\int_{0}^{t}\left(r_{1}\left(1-\frac{p\left(\tau \right)}{k_{1}}\right)+\frac{\alpha_{1}h\left(\tau \right)}{1+w+\left(\mathrm{ah}\left(\tau \right)+\mathrm{bp}\left(\tau \right)\right)}-\gamma_{1}\right)\mathrm{d\tau}}\geq 0$$

$$h\left(t\right)=h_{0}e^{\int_{0}^{t}\left(r_{2}\left(1-\frac{h\left(\tau \right)}{k_{2}}\right)+\frac{\alpha_{2}p\left(\tau \right)}{1+w+\left(\mathrm{ah}\left(\tau \right)+\mathrm{bp}\left(\tau \right)\right)}-\gamma_{2}\right)\mathrm{d\tau}}\geq 0$$

From the equation for honey production, we attain

$$\frac{\mathrm{dn}}{\mathrm{dt}}\geq -n(\beta h_{0}e^{\int_{0}^{t}\left(r_{2}\left(1-\frac{h\left(\tau \right)}{k_{2}}\right)+\frac{\alpha_{2}p\left(\tau \right)}{1+w+\left(\mathrm{ah}\left(\tau \right)+\mathrm{bp}\left(\tau \right)\right)}-\gamma_{2}\right)\mathrm{d\tau}}+\gamma_{3})\text{, which implies that}\;n\left(t\right)\geq n_{0}e^{\int_{0}^{t}\left(\beta h_{0}e^{\int_{0}^{t}\left(r_{2}\left(1-\frac{h\left(\tau \right)}{k_{2}}\right)+\frac{\alpha_{2}p\left(\tau \right)}{1+w+\left(\mathrm{ah}\left(\tau \right)+\mathrm{bp}\left(\tau \right)\right)}-\gamma_{2}\right)\mathrm{d\tau}}+\gamma_{3}\right)\mathrm{d\tau}}\geq 0$$

Therefore, the solution (p(t),h(t),n(t)) will remain positive for all t≥0 .

### Boundedness

Theorem 2. All solutions of phn system are uniformly bounded.

Proof. From the blooming plants equation of the phn system, we obtain

$$\frac{\mathrm{dp}}{\mathrm{dt}}=r_{1}p\left(1-\frac{p}{k_{1}}\right)+\frac{\alpha_{1}\mathrm{hp}}{1+w+\left(\mathrm{ah}+\mathrm{bp}\right)}-\gamma_{1}p\leq p\left[\left(r_{1}-\gamma_{1}\right)-\left(\frac{r_{1}p}{k_{1}}\right)+\alpha_{1}h\right]$$

After using the honeybees’ carrying capacity, we get

$$\frac{\mathrm{dp}}{\mathrm{dt}}\leq p\left[\left(r_{1}+\alpha_{1}k_{2}-\gamma_{1}\right)-\left(\frac{r_{1}p}{k_{1}}\right)\right]$$

Using Lemma 1, we deduced that.

$$p\left(t\right)\leq {\left[\frac{\left(r_{1}+\alpha_{1}k_{2}-\gamma_{1}\right)k_{1}}{r_{1}}\right]}^{\frac{1}{2}}{\left(1+\left[\frac{\left(r_{1}+\alpha_{1}k_{2}-\gamma_{1}\right)k_{1}p^{-2}\left(0\right)}{r_{1}}-1\right]e^{-2\left(r_{1}+\alpha_{1}k_{2}-\gamma_{1}\right)t}\right)}^{-\frac{1}{2}}$$

It is obtained after taking t→∞

$$p\left(t\right)\leq {\left[\frac{\left(r_{1}+\alpha_{1}k_{2}-\gamma_{1}\right)k_{1}}{r_{1}}\right]}^{\frac{1}{2}}=p_{m}$$

Similarly, for the honey honeybee equation of the phn system, we find

$$h\left(t\right)\leq {\left[\frac{k_{1}\left(r_{2}+\alpha_{2}p_{m}-\gamma_{2}\right)}{r_{2}}\right]}^{\frac{1}{2}}=h_{m}$$

Finally, from the amount of honey produced equation, we attain

$$\frac{\mathrm{dn}}{\mathrm{dt}}=\frac{\alpha_{3}h}{1+w+\mathrm{ch}}-\mathrm{\beta nh}-\gamma_{3}n\leq \alpha_{3}h_{m}-\gamma_{3}n$$

Then, by Lemma 2, we get

$$0\leq \underset{t\to \infty }{\text{lim}}\text{sup}\left[n\left(t\right)\right]\leq \frac{\alpha_{3}h_{m}}{\gamma_{3}}=n_{m}$$

Therefore, any solutions of phn system will be attracted to $\Sigma =\left\{\left(h,p,n\right)\in R_{+}^{3}:p\left(t\right)\leq p_{m},h\left(t\right)\leq h_{m},n\left(t\right)\leq n_{m}\right\}$ .

### Persistence

The phn system is said to be persistent if all its components survive in future times.

Theorem 3. All phn system components are persistent if

$$r_{1}>\gamma_{1},$$ (2)

$$r_{2}>\gamma_{2}.$$ (3)

Proof: First, to prove that p(t) If it is persistent, we have to show that $\lim_{t\to \infty }\inf p\left(t\right)>0$ i.e. p(t) will not decay to zero.

From the blooming plants equation, we get

$$\frac{\mathrm{dp}}{\mathrm{dt}}=r_{1}p\left(1-\frac{p}{k_{1}}\right)+\frac{\alpha_{1}\mathrm{hp}}{1+w+\left(\mathrm{ah}+\mathrm{bp}\right)}-\gamma_{1}p\geq p\left[\left(\;r_{1}-\gamma_{1}\right)-\left(\frac{r_{1}\;}{k_{1}}\right)p\right].$$

By Lemma 2, we have $\lim_{t\to \infty }\inf p\left(t\right)\geq L_{1}$ , where $L_{1}=\frac{k_{1}\left(r_{1}-\gamma_{1}\right)}{r_{1}}$ provided $r_{1}>\gamma_{1}$ . Thus, for a small $E_{1}>0$ , ∃ a positive number $t_{1}>0$ such that

$$p\left(t\right)\geq L_{1}-E_{1}\forall t>t_{1}$$

Applying the same strategy for the honeybee equation, we get

$$h\left(t\right)\geq L_{2}-E_{2}\forall t>t_{2},$$

where $L_{2}=\frac{k_{2}\left(r_{2}-\gamma_{2}\right)}{r_{2}}$ provided $r_{2}>\gamma_{2}$ .

From the honey production equation, we attain

$$\frac{\mathrm{dn}}{\mathrm{dt}}=\frac{\alpha_{3}h}{1+w+\mathrm{ch}}-\mathrm{\beta nh}-\gamma_{3}n\geq \frac{\alpha_{3}\left(L_{2}-E_{2}\right)}{1+w+c\left(L_{2}-E_{2}\right)}-n\left(\beta h_{m}-\gamma_{3}\right)$$

Since

•
$g\left(h\right)=\frac{\alpha_{3}h}{1+w+\mathrm{ch}}$ is an increasing function, which means if $h\left(t\right)\geq L_{2}-E_{2}$ , then $g\left(h\right)\geq g\left(L_{2}-E_{2}\right)$

•
$h\left(t\right)\leq h_{m}$

Then, by Lemma 2, $\lim_{t\to \infty }\inf n\left(t\right)\geq \frac{\alpha_{3}\left(L_{2}-E_{2}\right)}{\left[1+w+c\left(L_{2}-E_{2}\right)\right]\left(\beta h_{m}-\gamma_{3}\right)}$ , where $L_{3}=\frac{\alpha_{3}\left(L_{2}-E_{2}\right)}{\left[1+w+c\left(L_{2}-E_{2}\right)\right]\left(\beta h_{m}-\gamma_{3}\right)}$

Thus, for a small $E_{3}>0$ , ∃ a positive number $t_{3}>0$ such that

$$n\left(t\right)\geq L_{3}-E_{3}\forall t>t_{3}$$

Therefore, phn system is uniformly persistent.

Remark 1: Conditions 2 and 3 indicate that all phn system components will survive in the future times if the intrinsic growth rates of the blooming plants and honeybees exceed their depletion rates.

### Equilibrium analysis

The possible equilibria of phn system are

1) The extinction point $S_{1}=\left(0,0,0\right)$ .
2) The blooming plants point $S_{2}=\left(p_{1},0,0\right)$ , where $p_{1}=\frac{k_{1}\left(r_{1}-\gamma_{1}\right)}{r_{1}}$ . For $p_{1}$ to be positive, condition (2) must be satisfied.
3) The honeybee point $S_{3}=\left(0,h_{2},0\right)$ , where $h_{2}=\frac{k_{2}\left(r_{2}-\gamma_{2}\right)}{r_{2}}$ . Clearly $h_{2}>0$ if condition (3) is satisfied.
4) The blooming plants free point $S_{4}=\left(0,h_{3},n_{3}\right)$ , where $h_{3}=\frac{k_{2}\left(r_{2}-\gamma_{2}\right)}{r_{2}}$ and $n_{3}=\frac{\alpha_{3}h_{3}}{\left(1+w+ch_{3}\right)\left(\beta h_{3}+\gamma_{3}\right)}>0$ . Clearly $h_{3}>0$ if the condition (3) is satisfied.
5) The coexistence point $S_{5}=\left(p_{4},h_{4},n_{4}\right)$ , here $n_{4}=\frac{\alpha_{3}h_{4}}{\left(1+w+ch_{4}\right)\left(\beta h_{4}+\gamma_{3}\right)}$ , $h_{4}=\frac{r_{1}bp_{4}^{2}+z_{1}p_{4}+z_{2}}{z_{3}-ar_{1}p_{4}}\;$ where,

$z_{1}=r_{1}\left(1+w-k_{1}b\right)+\gamma_{1}k_{1}b,$

$z_{2}=k_{1}\left(\gamma_{1}\left(1+w\right)-\left(r_{1}+w\right)\right),$

$z_{3}=k_{1}\left(\alpha_{1}+a\left(r_{1}-\gamma_{1}-p\right)\right),$

and $p_{4}$ is the root of the following equation

$$A_{0}p^{3}+A_{1}P^{2}+A_{2}P+A_{3}=0,$$ (4)

where,

$A_{0}=r_{1}\left[r_{1}a\left(r_{2}b+r_{2}\mathrm{bw}+\alpha_{2}ak_{2}\right)-r_{2}b\left(bz_{3}+az_{1}\right)\right],$

$A_{1}=\left(r_{2}-\gamma_{2}\right)\left[r_{1}({\mathrm{ak}}_{2}\left(r_{1}+\mathrm{wa}r_{1}-bz_{3}-az_{1}\right)\right]+r_{2}\left(az_{1}+\mathrm{aw}z_{1}-\mathrm{bw}-\mathrm{bw}z_{3}-\mathrm{ab}z_{2}\right)-r_{2}bz_{1}z_{3}-r_{2}az_{1}^{2}$ $-ar_{1}\alpha_{2}z_{3}k_{2}-a\alpha_{2}r_{1}z_{3}k_{2},$

$A_{2}=\left(r_{2}-\gamma_{2}\right)\left[z_{3}k_{2}\left(z_{3}b-r_{1}-2r_{1}\mathrm{aw}+az_{1}-r_{1}a\right)-k_{2}r_{1}a^{2}z_{2}\right]+r_{2}(z_{2}\left(a\left(r_{1}-2z_{1}+wr_{1}\right)-bz_{3}\right)$ $+z_{3}\left(k_{2}\alpha_{2}-r_{1}-z_{1}w)\right),$

$A_{3}=\left(r_{2}-\gamma_{2}\right)\left[z_{3}\left(z_{3}-k_{2}\left(wz_{3}-az_{2}\right)\right)\right]-r_{2}z_{2}\left(z_{3}+wz_{3}+az_{2}\right).$

By Descartes’s rule of signs, Equation (4) has a unique positive root $p_{4}$ , if one of the following conditions holds:

$$\begin{array}{l} A_{0}>0,\text{and}\;A_{2},A_{3}<0,\\ A_{0},A_{1}>0,\text{and}\;A_{3}<0,\\ A_{0}<0,\text{and}\;A_{2},A_{3}>0,\\ A_{0},A_{1}<0,\text{and}\;A_{2},A_{3}>0. \end{array}$$

Further, $h_{4}>0$ if one of the following conditions holds:

$$\begin{array}{c} r_{1}bp_{4}^{2}+z_{1}p_{4}+z_{2}>0\;\text{and}\;z_{3}>ar_{1}p_{4}\\ r_{1}bp_{4}^{2}+z_{1}p_{4}+z_{2}<0\;\text{and}\;z_{3}<ar_{1}p_{4} \end{array}$$

### Local stability of equilibrium points

To investigate the local stability, one needs to determine the Jacobian matrix at any point. Thus, the Jacobian matrix at any point (p,h,n) is

$$J\left(p,h,n\right)=\left[\begin{array}{ccc} a_{11}\; & \frac{\alpha_{1}p\left(1+w+\mathrm{bp}\right)}{{\left(1+w+\left(\mathrm{ah}+\mathrm{bp}\right)\right)}^{2}} & 0\\ \frac{\alpha_{2}h\left(1+w+\mathrm{ah}\right)}{{\left(1+w+\left(\mathrm{ah}+\mathrm{bp}\right)\right)}^{2}} & a_{22} & 0\\ 0 & \frac{\alpha_{3}\left(1+w\right)}{{\left(1+w+\mathrm{ch}\right)}^{2}}-\mathrm{\beta n} & -\mathrm{\beta h}-\gamma_{3} \end{array}\right],$$

where $a_{11}=\frac{-2r_{1}p}{k_{1}}+\frac{\alpha_{1}h\left(1+w+\mathrm{ah}\right)}{{\left(1+w+\left(\mathrm{ah}+\mathrm{bp}\right)\right)}^{2}}+\left(r_{1}-\gamma_{1}\right)$ , and $a_{22}=\frac{-2r_{2}h}{k_{2}}+\frac{\alpha_{2}p\left(1+w+\mathrm{bp}\right)}{{\left(1+w+\left(\mathrm{ah}+\mathrm{bp}\right)\right)}^{2}}+\left(r_{2}-\gamma_{2}\right)$ .

Theorem 4. The extinction point $S_{1}=\left(0,0,0\right)$ is locally asymptotically stable if

$$\gamma_{1}>r_{1},$$ (5)

$$\gamma_{2}>r_{2}.$$ (6)

Proof: The Jacobian matrix at $S_{1}=\left(0,0,0\right)$ is

$$J\left(S_{1}\right)=\left[\begin{array}{ccc} r_{1}-\gamma_{1} & 0 & 0\\ 0 & r_{2}-\gamma_{2} & 0\\ 0 & \frac{\propto_{3}}{1+w} & -\gamma_{3} \end{array}\right],$$ (7)

Then, the eigenvalues of $J\left(S_{1}\right)$ are $\lambda_{11}=r_{1}-\gamma_{1}$ , $\lambda_{12}=r_{2}-\gamma_{2}$ and $\lambda_{13}=-\gamma_{3}<0$ .

Therefore, $S_{1}$ is asymptotically stable under conditions 5 and 6.

Remark 2: The biological interpretation of conditions 5 and 6 indicates phn system reaches asymptotically the extinction point when the depletion rates of the blooming plants and honeybees exceed their intrinsic growth rates.

Theorem 5. The blooming plants point $S_{2}=\left(p_{1},0,0\right)$ is locally asymptotically stable if

$$r_{2}+\frac{\alpha_{2}p_{1}}{\left(1+w+bp_{1}\right)}<\gamma_{2},$$ (8)

Proof:
$J\left(S_{2}\right)=J\left(p_{1},0,0\right)$ is given by

$$J\left(S_{2}\right)=\left[\begin{array}{ccc} -\left(r_{1}-\gamma_{1}\right)\; & \frac{\alpha_{1}p_{1}}{\left(1+w+bp_{1}\right)} & 0\\ 0 & \frac{\alpha_{2}p_{1}}{\left(1+w+bp_{1}\right)}+\left(r_{2}-\gamma_{2}\right) & 0\\ 0 & \frac{\alpha_{3}}{1+w} & -\gamma_{3} \end{array}\right].$$ (9)

So, the eigenvalues of $S_{2}$ are

$\lambda_{21}=-\left(r_{1}-\gamma_{1}\right)<0$ , under the existence condition of the blooming plants point.

$\lambda_{22}=\frac{\alpha_{2}p_{1}}{\left(1+w+bp_{1}\right)}+\left(r_{2}-\gamma_{2}\right),$ and $\;\lambda_{23}=-\gamma_{3}<0$ .

Thus, $S_{2}$ is asymptotically stable if condition 8 is satisfied .

Theorem 5. The honeybee point $\;S_{3}=\left(0,h_{2},0\right)$ is locally asymptotically stable if

$$r_{1}+\frac{\alpha_{1}h_{2}}{\left(1+w+ah_{2}\right)}<\gamma_{1},$$ (10)

$$\left(r_{2}-\gamma_{2}\right)<\frac{2r_{2}h_{2}}{k_{2}}$$ (11)

Proof:
$J\left(S_{3}\right)=J\left(0,h_{2},0\right)$ is given by

$$J\left(S_{3}\right)=\left[\begin{array}{ccc} \frac{\alpha_{1}h_{2}}{\left(1+w+ah_{2}\right)}+\left(r_{1}-\gamma_{1}\right)\; & 0 & 0\\ \frac{\alpha_{2}h_{2}}{1+w+ah_{2}} & \left(r_{2}-\gamma_{2}\right)-\frac{2r_{2}h_{2}}{k_{2}} & 0\\ 0 & \frac{\alpha_{3}\left(1+w\right)}{{\left(1+w+ch_{2}\right)}^{2}} & -\beta h_{2}-\gamma_{3} \end{array}\right].$$ (12)

So, the eigenvalues of $J\left(S_{3}\right)$ are $\lambda_{31}=\frac{\alpha_{1}h_{2}}{\left(1+w+ah_{2}\right)}+\left(r_{1}-\gamma_{1}\right),\lambda_{32}=\left(r_{2}-\gamma_{2}\right)-\frac{2r_{2}h_{2}}{k_{2}}$ , and $\lambda_{33}=-\beta h_{2}-\gamma_{3}<0$ . Thus, $S_{3}$ is asymptotically stable if the conditions 10 and 11 are satisfied.

Theorem 6. The blooming plants free point $S_{4}=\left(0,h_{3},n_{3}\right)\;$ is locally asymptotically stable if

$$r_{1}+\frac{\alpha_{1}h_{3}}{\left(1+w+ah_{3}\right)}<\gamma_{1},$$ (13)

$$\left(r_{2}-\gamma_{2}\right)<\frac{r_{2}h_{3}}{k_{2}}$$ (14)

Proof:
$J\left(S_{4}\right)=J\left(0,h_{3},n_{3}\right)$ is given by

$$J\left(S_{4}\right)=\left[\begin{array}{ccc} \frac{\alpha_{1}h_{3}}{\left(1+w+ah_{3}\right)}+\left(r_{1}-\gamma_{1}\right)\; & 0 & 0\\ \frac{\alpha_{2}h_{3}}{\left(1+w+ah_{3}\right)} & \left(r_{2}-\gamma_{2}\right)-\frac{2r_{2}h_{3}}{k_{2}} & 0\\ 0 & \frac{\alpha_{3}\left(1+w\right)}{\left(1+w+ch_{3}\right)}-\beta n_{3} & -\beta h_{3}-\gamma_{3} \end{array}\right].$$ (15)

The eigenvalues of $J\left(S_{4}\right)$ are $\lambda_{41}=\frac{\alpha_{1}h_{3}}{\left(1+w+ah_{3}\right)}+\left(r_{1}-\gamma_{1}\right)$ , $\lambda_{42}=\left(r_{2}-\gamma_{2}\right)-\frac{2r_{2}h_{3}}{k_{2}}$ , and $\lambda_{43}=-\beta h_{3}-\gamma_{3}<0$ . So, $S_{4}$ is asymptotically stable if conditions 13 and 14 are satisfied.

Theorem 7. The coexistence point $S_{5}=\left(p_{4},h_{4},n_{4}\right)$ is locally asymptotically stable if

$$a_{\mathrm{ii}}<0,i=1,2,$$ (16)

$$a_{11}a_{22}>\left[\frac{\alpha_{1}\alpha_{2}p_{4}h_{4}\left(1+w+bp_{4}\right)\left(1+w+ah_{4}\right)}{{\left(1+w+\left(ah_{4}+bp_{4}\right)\right)}^{4}}\right].$$ (17)

Proof:
$J\left(S_{5}\right)=J\left(p_{4},h_{4},n_{4}\right)$ is given by

$$J\left(S_{5}\right)=\left[\begin{array}{ccc} a_{11}\; & \frac{\alpha_{1}p_{4}\left(1+w+bp_{4}\right)}{{\left(1+w+\left(ah_{4}+bp_{4}\right)\right)}^{2}} & 0\\ \frac{\alpha_{2}h_{4}\left(1+w+ah_{4}\right)}{{\left(1+w+\left(ah_{4}+bp_{4}\right)\right)}^{2}} & a_{22} & 0\\ 0 & \frac{\alpha_{3}\left(1+w\right)}{{\left(1+w+ch_{4}\right)}^{2}}-\beta n_{4} & -\beta h_{4}-\gamma_{3} \end{array}\right],$$ (18)

where $a_{11}=\left(r_{1}-\gamma_{1}\right)-\frac{2r_{1}p_{4}}{k_{1}}+\frac{\alpha_{1}h_{4}\left(1+w+ah_{4}\right)}{{\left(1+w+\left(ah_{4}+bp_{4}\right)\right)}^{2}}$ , and $a_{22}=\left(r_{2}-\gamma_{2}\right)-\frac{2r_{2}h_{4}}{k_{2}}+\frac{\alpha_{2}p_{4}\left(1+w+bp_{4}\right)}{{\left(1+w+\left(ah_{4}+bp_{4}\right)\right)}^{2}}$ .

Then, the characteristic equation of $J\left(S_{5}\right)$ is given by:

$$\left(-\beta h_{4}-\gamma_{3}-\lambda \right)\left[\lambda^{2}-\mathrm{Tr\lambda}+\mathrm{Det}\right]=0,$$ (19)

where,

$$\begin{array}{l} \lambda_{51}=-\beta h_{4}-\gamma_{3},\\ \mathrm{Tr}=a_{11}+a_{22}=\left(r_{1}-\gamma_{1}\right)-\frac{2r_{1}p_{4}}{k_{1}}+\frac{\alpha_{1}h_{4}\left(1+w+ah_{4}\right)}{{\left(1+w+\left(ah_{4}+bp_{4}\right)\right)}^{2}}-\frac{2r_{2}h_{4}}{k_{2}}+\frac{\alpha_{2}p_{4}\left(1+w+bp_{4}\right)}{{\left(1+w+\left(ah_{4}+bp_{4}\right)\right)}^{2}}+\left(r_{2}-\gamma_{2}\right),\\ \mathrm{Det}=a_{11}a_{22}-\left[\frac{\alpha_{1}\alpha_{2}p_{4}h_{4}\left(1+w+bp_{4}\right)\left(1+w+ah_{4}\right)}{{\left(1+w+\left(ah_{4}+bp_{4}\right)\right)}^{4}}\right]=\left(\left(r_{1}-\gamma_{1}\right)-\frac{2r_{1}p_{4}}{k_{1}}+\frac{\alpha_{1}h_{4}\left(1+w+ah_{4}\right)}{{\left(1+w+\left(ah_{4}+bp_{4}\right)\right)}^{2}}\right)\left(\left(r_{2}-\gamma_{2}\right)-\frac{2r_{2}h_{4}}{k_{2}}+\frac{\alpha_{2}p_{4}\left(1+w+bp_{4}\right)}{{\left(1+w+\left(ah_{4}+bp_{4}\right)\right)}^{2}}\right)-\left[\frac{\alpha_{1}\alpha_{2}p_{4}h_{4}\left(1+w+bp_{4}\right)\left(1+w+ah_{4}\right)}{{\left(1+w+\left(ah_{4}+bp_{4}\right)\right)}^{4}}\right]. \end{array}$$

Thus, $S_{5}$ exhibits local stability if conditions 16 and 17 are fulfilled.

### Global stability

In this section, the Lyapunov method is used to illustrate the global stability of the previous points, as shown in the following theorems.

Theorem 8. The extinction point $S_{1}=\left(0,0,0\right)$ is a global asymptotic stability (GAS) if the following conditions are met.

$$r_{1}+\frac{h_{m}\left(\alpha_{1}+\alpha_{2}\right)}{1+w}<\gamma_{1}$$ (20)

$$r_{2}+\frac{\alpha_{3}}{1+w}<\gamma_{2}$$ (21)

Proof: Let $E_{1}\left(p,h,n\right)=p+h+n$ , where $E_{1}:R_{+}^{3}\to R$ , which satisfies $E_{1}\left(0,0,0\right)=0$ and $E_{1}\left(p,h,n\right)>0$ for all $\left(p,h,n\right)\in R_{+}^{3}$ with (p,h,n)≠(0,0,0) . Then

$$\frac{dE_{1}}{\mathrm{dt}}=\frac{\mathrm{dp}}{\mathrm{dt}}+\frac{\mathrm{dh}}{\mathrm{dt}}+\frac{\mathrm{dn}}{\mathrm{dt}}=\left(r_{1}p\left(1-\frac{p}{k_{1}}\right)+\frac{\alpha_{1}\mathrm{hp}}{1+w+\left(\mathrm{ah}+\mathrm{bp}\right)}-\gamma_{1}p\right)+\left(r_{2}h\left(1-\frac{h}{k_{2}}\right)+\frac{\alpha_{2}\mathrm{hp}}{1+w+\left(\mathrm{ah}+\mathrm{bp}\right)}-\gamma_{2}h\right)+\left(\frac{\alpha_{3}h}{1+w+\mathrm{ch})}-\mathrm{\beta nh}-\gamma_{3}n\right)=r_{1}p-r_{1}\frac{p^{2}}{k_{1}}+\frac{\mathrm{hp}\left(\alpha_{1}+\alpha_{2}\right)}{1+w+\left(\mathrm{ah}+\mathrm{bp}\right)}-\gamma_{1}p+r_{2}h-r_{2}\frac{h^{2}}{k_{2}}-\gamma_{2}h+\frac{\alpha_{3}h}{1+w+\mathrm{ch})}-\mathrm{\beta nh}-\gamma_{3}n$$

Then, by using the upper bound of the honeybees’ population, we get

$$\frac{dE_{1}}{\mathrm{dt}}\leq p\left(r_{1}-\gamma_{1}+\frac{h_{m}\left(\alpha_{1}+\alpha_{2}\right)}{1+w}\right)+h\left(r_{2}-\gamma_{2}+\frac{\alpha_{3}}{1+w}\right)-r_{1}\frac{p^{2}}{k_{1}}-r_{2}\frac{h^{2}}{k_{2}}-\mathrm{\beta nh}-\gamma_{3}n.$$

The first two terms are negative definite if conditions 20 and 21 are satisfied. Hence, $dE_{1}/\mathrm{dt}$ is a negative definite. Therefore, the extinction point $S_{1}$ is GAS.

Theorem 9. The blooming plants point $S_{2}=\left(p_{1},0,0\right)$ is GAS if

$$r_{2}+\frac{\alpha_{2}p_{m}+\alpha_{3}}{1+w}<\gamma_{2},$$ (22)

$${\left(\frac{\alpha_{1}}{D}\right)}^{2}\leq \frac{r_{1}r_{2}}{k_{1}k_{2}},$$ (23)

where, D=1+w+(ah+bp) .

Proof: Let $E_{2}=\left(p-p_{1}-p_{1}\ln\left(\frac{p}{p_{1}}\right)\right)+h+n$ , where $E_{2}:R_{+}^{3}\to R$ , which satisfies $E_{2}\left(p_{1},0,0\right)=0$ and $E_{2}\left(p,h,n\right)>0$ for all $\left(p,h,n\right)\in R_{+}^{3}$ with $\left(p,h,n\right)\neq \left(p_{1},0,0\right)$ , then

$$\frac{dE_{2}}{\mathrm{dt}}=\left(\frac{p-p_{1}}{p}\right)\frac{\mathrm{dp}}{\mathrm{dt}}+\frac{\mathrm{dh}}{\mathrm{dt}}+\frac{\mathrm{dn}}{\mathrm{dt}}=\frac{-r_{1}}{k_{1}}{\left(p-p_{1}\right)}^{2}+\frac{\alpha_{1}h\left(p-p_{1}\right)}{D}+hr_{2}\left(1-\frac{h}{k_{2}}\right)+\frac{\alpha_{2}\mathrm{ph}}{D}-\gamma_{2}h+\frac{\alpha_{3}h}{1+w+\mathrm{ch}}-\mathrm{\beta nh}-\gamma_{3}n$$

Then, by using the upper bound of the blooming plants population, we get

$$\frac{dE_{2}}{\mathrm{dt}}\leq -\left(\frac{r_{1}}{k_{1}}{\left(p-p_{1}\right)}^{2}-\frac{\alpha_{1}}{D}h\left(p-p_{1}\right)+\frac{r_{2}}{k_{2}}h^{2}\right)+h\left(r_{2}+\frac{\alpha_{2}p_{m}+\alpha_{3}}{1+w}-\gamma_{2}\right)-\mathrm{\beta nh}-\gamma_{3}n.$$

Thus,

$$\frac{dE_{2}}{\mathrm{dt}}\leq -{\left(\sqrt{\frac{r_{1}}{k_{1}}}\;\left(p-p_{1}\right)+\sqrt{\frac{r_{2}}{k_{2}}}\;h\right)}^{2}+h\left(r_{2}+\frac{\alpha_{2}p_{m}+\alpha_{3}}{1+w}-\gamma_{2}\right)-\mathrm{\beta nh}-\gamma_{3}n.$$

The first two terms are negative definite if conditions 22 and 23 are satisfied. Hence, $dE_{2}/\mathrm{dt}$ is a negative definite. Therefore, the blooming plants point $S_{2}=\left(p_{1},0,0\right)$ is GAS.

Theorem 10. The honeybee point $S_{3}=\left(0,h_{2},0\right)$ , is GAS if

$$r_{1}+\frac{\alpha_{1}\;h_{m}}{1+w}<\gamma_{1},$$ (24)

$${\left(\frac{\alpha_{2}}{D}\right)}^{2}\leq \frac{r_{1}r_{2}}{k_{1}k_{2}},$$ (25)

$$\frac{\alpha_{3}\;}{\beta \left(1+w\right)}<n,$$ (26)

where, D=1+w+(ah+bp) .

Proof: Let $E_{3}=p+\left(h-h_{2}-h_{2}\ln\frac{h}{h_{2}}\right)+n$ , where $E_{3}:R_{+}^{3}\to R$ , which satisfies $E_{3}\left(0,h_{2},0\right)=0$ and $E_{3}\left(p,h,n\right)>0$ for all $\left(p,h,n\right)\in R_{+}^{3}$ with $\left(p,h,n\right)\neq \left(0,h_{2},0\right)$ , then

$$\frac{dE_{3}}{\mathrm{dt}}=\frac{\mathrm{dp}}{\mathrm{dt}}+\left(\frac{h-h_{2}}{h}\right)\frac{\mathrm{dh}}{\mathrm{dt}}+\frac{\mathrm{dn}}{\mathrm{dt}}=\left(r_{1}p\left(1-\frac{p}{k_{1}}\right)+\frac{\alpha_{1}\mathrm{hp}}{1+w+\left(\mathrm{ah}+\mathrm{bp}\right)}-\gamma_{1}p\;\right)+\left(h-h_{2}\right)\left((r_{2}\left(1-\frac{h}{k_{2}}\right)+\frac{\alpha_{2}p}{1+w+\left(\mathrm{ah}+\mathrm{bp}\right)}-\gamma_{2})\right)+\left(\frac{\alpha_{3}h}{1+w+\mathrm{ch}}-\mathrm{\beta nh}-\gamma_{3}n\right)\leq r_{1}p-\frac{r_{1}p^{2}}{k_{1}}+\frac{\alpha_{1}\mathrm{hp}}{1+w}-\gamma_{1}p-\frac{r_{2}}{k_{2}}{\left(h-h_{2}\right)}^{2}+\frac{\alpha_{2}\left(h-h_{2}\right)p}{D}+\frac{\alpha_{3}h}{1+w}-\mathrm{\beta nh}-\gamma_{3}n$$

Then, by using the upper bound of the honeybees’ population, we get

$$\frac{dE_{3}}{\mathrm{dt}}\leq -\left(\frac{r_{1}}{k_{1}}p^{2}-\frac{\alpha_{2}\left(h-h_{2}\right)p}{D}+\left(\frac{r_{2}}{k_{2}}\right){\left(h-h_{2}\right)}^{2}\right)+p\left(r_{1}+\frac{\alpha_{1}\;h_{m}}{1+w}-\gamma_{1}\right)+h\left(\frac{\alpha_{3}\;}{1+w}-\mathrm{\beta n}\right)-\gamma_{3}n$$

Thus,

$$\frac{dE_{3}}{\mathrm{dt}}\leq -{\left(\sqrt{\frac{r_{1}}{k_{1}}p}+\sqrt{\frac{r_{2}}{k_{2}}\;}\left(h-h_{2}\right)\right)}^{2}+p\left(r_{1}+\frac{\alpha_{1}\;h_{m}}{1+w}-\gamma_{1}\right)+h\left(\frac{\alpha_{3}\;}{1+w}-\mathrm{\beta n}\right)-\gamma_{3}n.$$

The first three terms are negative definite if conditions 24-26 are satisfied. Hence, $dE_{3}/\mathrm{dt}$ is a negative definite. Therefore, the blooming plants point $S_{3}=\left(0,h_{2},0\right)$ is GAS.

The blooming plants free point $S_{4}=\left(0,h_{3},n_{3}\right)$

Theorem 11. The blooming plants free point $S_{4}=\left(0,h_{3},n_{3}\right)$ is GAS if

$${\left(\frac{\alpha_{2}}{D}\right)}^{2}\leq \frac{r_{1}r_{2}}{2k_{1}k_{2}},$$ (27)

$${\left(\frac{\alpha_{3}\left(1+w\right)}{D_{1}D_{2}}-\mathrm{\beta n}\right)}^{2}\leq \frac{r_{2}\left(\beta h_{3}+\gamma_{3}\right)}{2k_{2}},$$ (28)

where, $D=1+w+\left(\mathrm{ah}+\mathrm{bp}\right),D_{1}=\left(1+w+\mathrm{ch}\right)$ and $D_{2}=\left(1+w+ch_{3}\right)$ .

Proof: Let $E_{4}=p+\left(h-h_{3}-h_{3}\ln\frac{h}{h_{3}}\right)+{\left(\frac{n-n_{3}}{2}\right)}^{2}$ , where $E_{4}:R_{+}^{3}\to R$ , which satisfies $E_{4}\left(0,h_{3},n_{3}\right)=0$ and $E_{4}\left(p,h,n\right)>0$ for all $\left(p,h,n\right)\in R_{+}^{3}$ with $\left(p,h,n\right)\neq \left(0,h_{3},n_{3}\right)$ , then

$$\frac{dE_{4}}{\mathrm{dt}}=\frac{\mathrm{dp}}{\mathrm{dt}}+\left(\frac{h-h_{3}}{h}\right)\frac{\mathrm{dh}}{\mathrm{dt}}+\left(n-n_{3}\right)\frac{\mathrm{dn}}{\mathrm{dt}}=\left(r_{1}p\left(1-\frac{p}{k_{1}}\right)+\frac{\alpha_{1}\mathrm{hp}}{1+w+\left(\mathrm{ah}+\mathrm{bp}\right)}-\gamma_{1}p\;\right)+\left(h-h_{3}\right)\left(r_{2}\left(1-\frac{h}{k_{2}}\right)+\frac{\alpha_{2}p}{1+w+\left(\mathrm{ah}+\mathrm{bp}\right)}-\gamma_{2}\right)+\left(n-n_{3}\right)\left(\frac{\alpha_{3}h}{1+w+\mathrm{ch}}-\mathrm{\beta nh}-\gamma_{3}n\right)=r_{1}p-\frac{r_{1}p^{2}}{k_{1}}+\frac{\alpha_{1}\mathrm{hp}}{1+w+\left(\mathrm{ah}+\mathrm{bp}\right)}-\gamma_{1}p+\left(h-h_{3}\right)\left(-\frac{r_{2}}{k_{2}}\left(h-h_{3}\right)+\frac{\alpha_{2}p}{1+w+\left(\mathrm{ah}+\mathrm{bp}\right)}\right)+\left(n-n_{3}\right)\left(\frac{\alpha_{3}h}{1+w+\mathrm{ch}}-\frac{\alpha_{3}h_{3}}{1+w+ch_{3}}-\mathrm{\beta nh}-\beta n_{3}h_{3}-\gamma_{3}\left(n-n_{3}\right)\right)$$

Then, by using the upper bound of the honeybees’ population, we get

$$\frac{dE_{4}}{\mathrm{dt}}\leq -\left(\frac{r_{1}}{k_{1}}p^{2}-\frac{\alpha_{2}\left(h-h_{3}\right)p}{1+w+\left(\mathrm{ah}+\mathrm{bp}\right)}+\left(\frac{r_{2}}{2k_{2}}\right){\left(h-h_{3}\right)}^{2}\right)+p\left(r_{1}+\frac{\alpha_{1}h_{m}}{1+w}-\gamma_{1}\right)-\left[\left(\frac{r_{2}}{2k_{2}}\right){\left(h-h_{3}\right)}^{2}-\left(\frac{\alpha_{3}\left(1+w\right)}{\left(1+w+\mathrm{ch}\right)\left(1+w+ch_{3}\right)}-\mathrm{\beta n}\right)\left(h-h_{3}\right)\left(n-n_{3}\right)+\left(\beta h_{3}+\gamma_{3}\right){\left(n-n_{3}\right)}^{2}\right]$$

Thus,

$$\frac{dE_{4}}{\mathrm{dt}}\leq -{\left(\sqrt{\frac{r_{1}}{k_{1}}p}+\sqrt{\frac{r_{2}}{2k_{2}}\;}\left(h-h_{3}\right)\right)}^{2}+p\left(r_{1}+\frac{\alpha_{1}h_{m}}{1+w}-\gamma_{1}\right)-{\left(\sqrt{\frac{r_{2}}{2k_{2}}}\left(h-h_{3}\right)+\sqrt{\left(\beta h_{3}+\gamma_{3}\right)}\left(n-n_{3}\right)\right)}^{2}$$

The first and the third terms are negative definite if conditions 27 and 28 are satisfied, while the second term is negative under condition 24. Hence, $dE_{4}/\mathrm{dt}$ is a negative definite. Therefore, the blooming plants free point $S_{4}=\left(0,h_{3},n_{3}\right)$ is GAS.

Theorem 12. The coexistence point $S_{5}=\left(p_{4},h_{4},n_{4}\right)$ is GAS if

$${\left(\frac{\left(\alpha_{1}+\alpha_{2}\right)\left(1+w\right)+\alpha_{1}bp_{4}+\alpha_{2}ah_{4}}{N_{1}N_{2}}\right)}^{2}\leq \frac{1}{2}\left(\frac{r_{1}}{k_{1}}+\frac{\alpha_{1}bh_{4}}{N_{1}N_{2}}\right)\left(\frac{r_{2}}{k_{2}}+\frac{\alpha_{2}ap_{4}}{N_{1}N_{2}}\right),$$ (29)

$${\left(\frac{\alpha_{3}\left(1+w\right)}{D_{1}D_{2}}-\mathrm{\beta n}\right)}^{2}\leq \frac{1}{2}\left(\frac{r_{2}}{k_{2}}+\frac{\alpha_{2}ap_{4}}{N_{1}N_{2}}\right)\left(\beta h_{4}+\gamma_{3}\right),$$ (30)

where, $N_{1}=1+w+\left(\mathrm{ah}+\mathrm{bp}\right)$ , $N_{2}=1+w+\left(ah_{4}+bp_{4}\right),D_{1}=\left(1+w+\mathrm{ch}\right)$ and $D_{2}=\left(1+w+ch_{3}\right)$ .

Proof: Let $E_{5}=\left(p-p_{4}-p_{4}\ln\frac{p}{p_{4}}\right)+\left(h-h_{4}-h_{4}\ln\frac{h}{h_{4}}\right)+{\left(\frac{n-n_{4}}{2}\right)}^{2}$ , where $E_{5}:R_{+}^{3}\to R$ , which satisfies $E_{5}\left(p_{4},h_{4},n_{4}\right)=0$ and $E_{5}\left(p,h,n\right)>0$ for all $\left(p,h,n\right)\in R_{+}^{3}$ with $\left(p,h,n\right)\neq \left(p_{4},h_{4},n_{4}\right)$ , then

$$\frac{dE_{5}}{\mathrm{dt}}=\left(\frac{p-p_{4}}{p}\right)\frac{\mathrm{dp}}{\mathrm{dt}}+\left(\frac{h-h_{4}}{h}\right)\frac{\mathrm{dh}}{\mathrm{dt}}+\left(n-n_{4}\right)\frac{\mathrm{dn}}{\mathrm{dt}}=\left(p-p_{4}\right)\left(r_{1}\left(1-\frac{p}{k_{1}}\right)+\frac{\alpha_{1}h}{1+w+\left(\mathrm{ah}+\mathrm{bp}\right)}-\gamma_{1}\;\right)+\left(h-h_{4}\right)\left(r_{2}\left(1-\frac{h}{k_{2}}\right)+\frac{\alpha_{2}p}{1+w+\left(\mathrm{ah}+\mathrm{bp}\right)}-\gamma_{2}\right)+\left(n-n_{4}\right)\left(\frac{\alpha_{3}h}{1+w+\mathrm{ch}}-\mathrm{\beta nh}-\gamma_{3}n\right)\frac{dE_{5}}{\mathrm{dt}}=-\left[\left(\frac{r_{1}}{k_{1}}+\frac{\alpha_{1}bh_{4}}{N_{1}N_{2}}\right){\left(p-p_{4}\right)}^{2}-\left(\frac{\left(\alpha_{1}+\alpha_{2}\right)\left(1+w\right)+\alpha_{1}bp_{4}+\alpha_{2}ah_{4}}{N_{1}N_{2}}\right)\left(p-p_{4}\right)\left(h-h_{4}\right)+\frac{1}{2}\left(\frac{r_{2}}{k_{2}}+\frac{\alpha_{2}ap_{4}}{N_{1}N_{2}}\right){\left(h-h_{4}\right)}^{2}\right]-\left[\frac{1}{2}\left(\frac{r_{2}}{k_{2}}+\frac{\alpha_{2}ap_{4}}{N_{1}N_{2}}\right){\left(h-h_{4}\right)}^{2}-\left(\frac{\alpha_{3}\left(1+w\right)}{D_{1}D_{2}}-\mathrm{\beta n}\right)\left(n-n_{4}\right)\left(h-h_{4}\right)+\left(\beta h_{4}+\gamma_{3}\right){\left(n-n_{4}\right)}^{2}\right]$$

Thus,

$$\frac{dE_{5}}{\mathrm{dt}}\leq -{\left(\sqrt{\left(\frac{r_{1}}{k_{1}}+\frac{\alpha_{1}bh_{4}}{N_{1}N_{2}}\right)}\left(p-p_{4}\right)+\sqrt{\frac{1}{2}\left(\frac{r_{2}}{k_{2}}+\frac{\alpha_{2}ap_{4}}{N_{1}N_{2}}\right)}\left(h-h_{4}\right)\right)}^{2}-{\left(\sqrt{\frac{1}{2}\left(\frac{r_{2}}{k_{2}}+\frac{\alpha_{2}ap_{4}}{N_{1}N_{2}}\right)}\left(h-h_{4}\right)+\sqrt{\left(\beta h_{4}+\gamma_{3}\right)}\left(n-n_{4}\right)\right)}^{2}$$

Hence, $dE_{5}/\mathrm{dt}$ is a negative definite under conditions 29 and 30. Therefore, the coexistence point $S_{5}=\left(p_{4},h_{4},n_{4}\right)$ is GAS.

### Bifurcation

This section explores the probability of occurrence of transcritical (TB) and Hopf bifurcation (HB) around the non-hyperbolic equilibrium points. For more details, see Refs. 23- 26.

Theorem 13. For $r_{2}^{*}=\gamma_{2}$ , the phn model faces TB at the extinction point $S_{1}=\left(0,0,0\right)$ .

Proof: According to $J\left(S_{1}\right)$ given by Eq. (7), the phn system at $S_{1}$ has a zero eigenvalue $\lambda_{12}=0$ , at $r_{2}^{*}=\gamma_{2}$ , and $J\left(S_{1}\right)$ at $r_{2}^{*}=\gamma_{2}$ becomes

$$J^{*}\left(S_{1}\right)=\left[\begin{array}{ccc} r_{1}-\gamma & 0 & 0\\ 0 & 0 & 0\\ 0 & \frac{\alpha_{3}}{1+w} & -\gamma_{3} \end{array}\right]$$

Now, suppose that $\vartheta^{\left[1\right]}={\left(\vartheta_{1}^{\left[1\right]},\vartheta_{2}^{\left[1\right]},\vartheta_{3}^{\left[1\right]}\right)}^{T}$ , and ${\left(T^{\left[1\right]}\right)}^{T}={\left(t_{1}^{\left[1\right]},t_{2}^{\left[1\right]},t_{3}^{\left[1\right]}\right)}^{T}$ be eigenvectors to $\lambda_{22}=0$ of $J^{*}\left(S_{1}\right)$ , and $J^{*T}\left(S_{1}\right)$ , respectively. The calculation gives $\vartheta^{\left[1\right]}=(0,1,\frac{\alpha_{3}}{\gamma_{3}\left(1+w\right)}$ ), and ${\left(T^{\left[1\right]}\right)}^{T}=\left(0,1,0\right)$ by solving $\left(J^{*}\left(S_{1}\right)-\lambda_{12}I\right)\vartheta^{\left[1\right]}$ , and $\;\left(J^{*T}\left(S_{1}\right)-\lambda_{12}I\right)T^{\left[1\right]}$ for $\vartheta^{\left[1\right]}$ and $T^{\left[1\right]}$ .

Further,

$$F_{r_{2}}\left(S,r_{2}\right)=\left(0,h\left(1-\frac{h}{k_{2}}\right),0\right)\Rightarrow F_{r_{2}}\left(S_{1},r_{2}^{*}\right)=\left(0,0,0\right)$$

$${\left(T^{\left[1\right]}\right)}^{T}F_{r_{2}}\left(S_{1},r_{2}^{*}\right)=\left(0,1,0\right)\left(0,0,0\right)=0$$

$${\left(T^{\left[1\right]}\right)}^{T}DF_{r_{2}}\left(S_{1},r_{2}^{*}\right)\vartheta^{\left[1\right]}=\left(0,1,0\right)\left[\begin{array}{ccc} 0 & 0 & 0\\ 0 & 1 & 0\\ 0 & 0 & 0 \end{array}\right]=1\neq 0$$

$${\left(T^{\left[1\right]}\right)}^{T}D^{2}F_{r_{2}}\left(S_{1},r_{2}^{*}\right)\left(\vartheta^{\left[1\right]},\vartheta^{\left[1\right]}\right)=\left(0,1,0\right)\left[\begin{array}{c} 0\\ \frac{-2r_{2}^{*}}{k_{2}}\\ 0 \end{array}\right]=\frac{-2r_{2}^{*}}{k_{2}}\neq 0$$

Therefore, there is a TB around $S_{1}$ with the parameter $r_{2}^{*}=\gamma_{2}$ .

Theorem 14. For $r_{1}^{*}=\gamma_{1}$ , the phn model faces TB at the extinction point $S_{2}=\left(p_{1},0,0\right)$ if

$$p_{1}=k_{1},$$ (30)

$${\left(T^{\left[2\right]}\right)}^{T}D^{2}F_{r_{1}}\left(S_{2},r_{1}^{*}\right)\left(\vartheta^{\left[2\right]},\vartheta^{\left[2\right]}\right)\neq 0$$ (31)

Proof: According to $J\left(S_{2}\right)$ given by Eq. (9), the phn system at $S_{2}$ has a zero eigenvalue $\lambda_{22}=0$ , at $r_{1}^{*}=\gamma_{1}$ , and $J\left(S_{2}\right)$ at $r_{1}^{*}=\gamma_{1}$ becomes

$$J^{*}\left(S_{2}\right)=\left[\begin{array}{ccc} 0 & 0 & 0\\ 0 & r_{2}-\gamma_{2} & 0\\ 0 & \frac{\alpha_{3}}{1+w} & -\gamma_{3} \end{array}\;\right],$$

Now, suppose that $\vartheta^{\left[2\right]}={\left(\vartheta_{1}^{\left[2\right]},\vartheta_{2}^{\left[2\right]},\vartheta_{3}^{\left[2\right]}\right)}^{T}$ and ${\left(T^{\left[1\right]}\right)}^{T}={\left(t_{1}^{\left[2\right]},t_{2}^{\left[2\right]},t_{3}^{\left[2\right]}\right)}^{T}$ be eigenvectors with respect to $\lambda_{22}=0$ of $J^{*}\left(S_{2}\right)$ and ${J^{*}}^{T}\left(S_{2}\right)$ respectively. Solving $\left(J^{*}\left(S_{2}\right)-\lambda_{22}I\right)\vartheta^{\left[2\right]}=0$ , and $\;\left(J^{*T}\left(S_{2}\right)-\lambda_{22}I\right)T^{\left[2\right]}=0$ for $\vartheta^{\left[2\right]}$ , and $T^{\left[2\right]}$ gives $\vartheta^{\left[2\right]}={\left(1,1,\frac{\alpha_{3}}{\gamma_{3\left(1+w\right)}}\right)}^{T}$ and ${\left(T^{\left[2\right]}\right)}^{T}={\left(1,\frac{\alpha_{3}}{\left(1+w\right)\left(\gamma_{2}-r_{2}\right)},1\right)}^{T}$ , where $\left(\gamma_{2}-r_{2}\right)\neq 0$ .

Further,

$$F_{r_{1}}\left(S,r_{1}\right)=\left(p\left(1-\frac{p}{k_{1}}\right),0,0\right)⟹F_{r_{1}}\left(S_{2},r_{1}^{*}\right)=\left(p_{1}\left(1-\frac{p_{1}}{k_{1}}\right),0,0\right)$$

$${\left(T^{\left[2\right]}\right)}^{T}F_{r_{1}}\left(S_{2},r_{1}^{*}\right)=\left(1,\frac{\propto_{3}}{\left(1+w\right)\left(\gamma_{2}-r_{2}\right)},1\right)\left(p_{1}-\frac{p_{1}^{2}}{k_{1}},0,0\right)=p_{1}\left(1-\frac{p_{1}}{k_{1}}\right)=0\;\text{under condition}\;30.$$

$${\left(T^{\left[2\right]}\right)}^{T}{\mathrm{DF}}_{r_{1}}\left(S_{2},r_{1}^{*}\right)\left(\vartheta^{\left[2\right]}\right)=\left(1,\frac{\alpha_{3}}{\left(1+w\right)\left(\gamma_{2}-r_{2}\right)},1\right)\left[\begin{array}{ccc} 1 & 0 & 0\\ 0 & 0 & 0\\ 0 & 0 & 0 \end{array}\right]=1\neq 0$$

${\left(T^{\left[2\right]}\right)}^{T}D^{2}F_{r_{1}}\left(S_{2},r_{1}^{*}\right)\left(\vartheta^{\left[2\right]},\vartheta^{\left[2\right]}\right)=\left(1,\frac{\alpha_{3}}{\left(1+w\right)\left(\gamma_{2}-r_{2}\right)},1\right)\left[\begin{array}{c} X_{11}^{\left[2\right]}+X_{12}^{\left[2\right]}\\ X_{21}^{\left[2\right]}+X_{22}^{\left[2\right]}\\ X_{32}^{\left[2\right]}+X_{33}^{\left[2\right]}\frac{\propto_{3}}{\gamma_{3}\left(1+w\right)} \end{array}\right]$

$=\left(X_{11}^{\left[2\right]}+X_{12}^{\left[2\right]}\right)+\frac{\alpha_{3}}{\left(1+w\right)\left(\gamma_{2}-r_{2}\right)}\left(X_{21}^{\left[2\right]}+X_{22}^{\left[2\right]}\right)+\left(X_{32}^{\left[2\right]}+X_{33}^{\left[2\right]}\frac{\alpha_{3}}{\gamma_{3}\left(1+w\right)}\right)\neq 0$

under condition 31, here, $X_{11}^{\left[2\right]}=\frac{-2r_{1}}{k_{1}}+\left[\frac{\alpha_{1}\left[(1+w)\right]}{{\left(1+w+bp_{1}\right)}^{2}}\right]$ , $X_{12}^{\left[2\right]}=\left[\frac{\alpha_{1}\left(1+w\right)}{{\left(1+w+bp_{1}\right)}^{2}}\right]+\left[\frac{-2a\alpha_{1}p_{1}}{{\left(1+w+bp_{1}\right)}^{2}}\right]$ , $X_{21}^{\left[2\right]}=[\frac{\alpha_{2}\left(1+w\right)}{{\left(1+w+bp_{1}\right)}^{2}}]$ , $X_{22}^{\left[2\right]}=\left[\frac{\alpha_{2}\left[(1+w)\right]}{{\left(1+w+bp_{1}\right)}^{2}}\right]+\left[\frac{-2r_{2}}{k_{2}}-\frac{2a\alpha_{2}p_{1}}{{\left(1+w+bp_{1}\right)}^{2}}\right]$ , $X_{32}^{\left[2\right]}=\frac{-2c\alpha_{3}}{{\left(1+w\right)}^{2}}-\frac{\beta \alpha_{3}}{\gamma_{3}\left(1+w\right)},\text{and}\;X_{33}^{\left[2\right]}=\frac{-\beta \alpha_{3}}{\gamma_{3}\left(1+w\right)}.$

Therefore, there is a TB around $S_{2}$ with the parameter $r_{1}^{*}=\gamma_{1}$ .

Theorem 15. For ${r_{2}}^{**}=\frac{\gamma_{2}k_{2}}{k_{2}-2h_{2}}$ , the phn model faces TB at the honeybee point $S_{3}=\left(0,h_{2},0\right)$ if

$$h_{2}=k_{2},$$ (32)

Proof: According to $J\left(S_{3}\right)$ given by Eq. (11), the phn system at $S_{3}$ has a zero eigenvalue $\lambda_{32}=0$ , at ${r_{2}}^{**}$ , and $J\left(S_{3}\right)$ at ${r_{2}}^{**}$ becomes

$$J^{*}\left(S_{3}\right)=\left[\begin{array}{ccc} \frac{\alpha_{1}h_{2}}{1+w+ah_{2}}+\left(r_{1}-\gamma_{1}\right) & 0 & 0\\ \frac{\alpha h_{2}}{1+w+ah_{2}} & 0 & 0\\ 0 & \frac{\left(1+w\right)\alpha_{3}}{{\left(1+w+ch_{2}\right)}^{2}} & -\left(\beta h_{2}+\gamma_{3}\right) \end{array}\right]$$

Now, Suppose that $\vartheta^{\left[3\right]}=\left(\vartheta_{1}^{\left[3\right]},\vartheta_{2}^{\left[3\right]},\vartheta_{3}^{\left[3\right]}\right)$ and ${\left(T^{\left[3\right]}\right)}^{T}={\left(t_{1}^{\left[3\right]},t_{2}^{\left[3\right]},t_{3}^{\left[3\right]}\right)}^{T}$ be eigenvectors to $\lambda_{32}=0$ of $J^{*}\left(S_{3}\right)$ and ${J^{*}}^{T}\left(S_{3}\right)$ respectively. Solving $\left(J^{*}\left(S_{3}\right)-\lambda_{32}I\right)\vartheta^{\left[3\right]}=0$ , and $\;\left(J^{*T}\left(S_{3}\right)-\lambda_{32}I\right)T^{\left[3\right]}=0$ for $\vartheta^{\left[3\right]}$ and $T^{\left[3\right]}$ gives $V^{\left[3\right]}=\left(0,1,\frac{\alpha_{3}\left(1+w\right)}{\left(\beta h_{2}+\gamma_{3}\right){\left(1+w+ch_{2}\right)}^{2}}\right)$ and ${\left(T^{\left[3\right]}\right)}^{T}=\left(\frac{-\alpha_{2}h_{2}}{\left(\alpha_{1}h_{2}+\left(r_{1}-\gamma_{1}\right)\left(1+w+ah_{2}\right)\right)},1,0\right)$ , where $[\alpha_{1}h_{2}+\left[\left(r_{1}-\gamma_{1}\right)\left(1+w+ah_{2}\right)\right]\neq 0$ .

Further

$$\begin{array}{c} F_{r_{2}}\left(S_{3},r_{2}\right)=\left(0,h-\frac{h}{k_{2}},0\right)\Rightarrow F_{r_{2}}\left(S_{3},r_{2}^{**}\right)=\left(0,h_{2}\left(1-\frac{h_{2}}{k_{2}}\right),0\right)\\ {\left(T^{\left[3\right]}\right)}^{T}F_{r_{2}}\left(S_{3},r_{2}^{**}\right)=\left(\frac{-\alpha_{2}h_{2}}{\left(\alpha_{1}h_{2}+\left(r_{1}-\gamma_{1}\right)\left(1+w+ah_{2}\right)\right)},1,0\right)\left(0,h_{2}\left(1-\frac{h_{2}}{k_{2}}\right),0\right)\\ \begin{array}{c} {\left(T^{\left[3\right]}\right)}^{T}F_{r_{2}}\left(S_{3},r_{2}^{**}\right)=h_{2}\left(1-\frac{h_{2}}{k_{2}}\right)=0\;\text{under condition}\;32.\\ \begin{array}{c} {\left(T^{\left[3\right]}\right)}^{T}{\mathrm{DF}}_{r_{2}}\left(S_{3},r_{2}^{**}\right)\left(\vartheta^{\left[3\right]}\right)=\left(\frac{-\alpha_{2}h_{2}}{\left(\alpha_{1}h_{2}+\left(r_{1}-\gamma_{1}\right)\left(1+w+ah_{2}\right)\right)},1,0\right)\left[\begin{array}{ccc} 0 & 0 & 0\\ 0 & 1 & 0\\ 0 & 0 & 0 \end{array}\right]=1\neq 0\\ {\left(T^{\left[3\right]}\right)}^{T}D^{2}F_{r_{2}}\left(S_{3},r_{2}^{**}\right)\left(\vartheta^{\left[3\right]},\vartheta^{\left[3\right]}\right)=\left(\frac{-\alpha_{2}h_{2}}{\left(\alpha_{1}h_{2}+\left(r_{1}-\gamma_{1}\right)\left(1+w+ah_{2}\right)\right)},1,0\right)\left[\begin{array}{c} 0\\ \frac{-2r_{2}^{**}}{k_{2}}\\ X_{32}^{\left[3\right]}-\beta \frac{\alpha_{3}\left(1+w\right)}{\left(\beta h_{2}+\gamma_{2}\right){\left(1+w+ch_{2}\right)}^{2}} \end{array}\right]=\frac{-2r_{2}^{**}}{k_{2}}\neq 0, \end{array} \end{array} \end{array}$$

where $\;X_{32}^{\left[3\right]}=\frac{-2c\alpha_{3}\left(1+w\right)}{{\left(1+w+ch_{2}\right)}^{3}}-\frac{\beta \alpha_{3}\left(1+w\right)}{\left(\beta h_{2}+\gamma_{2}\right){\left(1+w+ch_{2}\right)}^{2}}$

Therefore, there is a TB around $S_{3}$ with the parameter $r_{2}^{**}$ .

Theorem 16. For ${r_{2}}^{***}=\frac{\gamma_{2}k_{2}}{k_{2}-2h_{3}}$ , the phn model faces TB at the honeybee point $S_{4}=\left(0,h_{3},n_{3}\right)$ if

$$h_{3}=k_{2},$$ (33)

Proof: According to $J\left(S_{4}\right)$ given by Eq. (15), the phn system at $S_{4}$ has a zero eigenvalue $\lambda_{42}=0$ , at ${r_{2}}^{***}$ , and $J\left(S_{4}\right)$ at ${r_{2}}^{***}$ becomes

$$J^{*}\left(S_{4}\right)=\left[\begin{array}{ccc} a_{11}\; & 0 & 0\\ a_{21} & 0 & 0\\ 0 & a_{32} & -\beta h_{3}-\gamma_{3} \end{array}\right],$$

where, $a_{11}=\frac{\alpha_{1}h_{3}}{\left(1+w+ah_{3}\right)}+\left(r_{1}-\gamma_{1}\right)$ , $a_{21}=\frac{\alpha_{2}h_{3}}{1+w+ah_{3}}$ and $a_{32}=\frac{\alpha_{3}\left(1+w\right)}{\left(1+w+ch_{3}\right)}-\beta n_{3}$ .

Now, Suppose that $\vartheta^{\left[4\right]}=\left(\vartheta_{1}^{\left[4\right]},\vartheta_{2}^{\left[4\right]},\vartheta_{3}^{\left[4\right]}\right)$ , and ${\left(T^{\left[4\right]}\right)}^{T}=\left(t_{1}^{\left[4\right]},t_{2}^{\left[4\right]},t_{3}^{\left[4\right]}\right)$ be an eigenvector of to $\lambda_{42}=0$ of $J^{*}\left(S_{4}\right)$ and $J^{*T}\left(S_{4}\right)$ , respectively, which gives $\vartheta^{\left[4\right]}=\left(0,1,\frac{a_{32}}{\beta h_{3}+\gamma_{3}}\right)$ and ${\left(T^{\left[4\right]}\right)}^{T}=\left(\frac{-a_{21}}{a_{11}},1,0\right)$ .

Further,

$$\begin{array}{c} F_{r_{2}}\left(S,r_{2}\right)=\left(0,h\left(1-\frac{h}{k_{2}}\right),0\right)\Rightarrow F_{r_{2}}\left(S_{4},r_{2}^{***}\right)=\left(0,h_{2}-\frac{h_{2}}{k_{2}},0\right)\\ {\left(T^{\left[4\right]}\right)}^{T}F_{r_{2}}\left(S_{3},r_{2}^{*}\right)=\left(\frac{-a_{21}}{a_{11}},1,0\right)\left(0,h_{3}^{2}-\frac{h_{3}^{2}}{k_{2}},0\right)=h_{3}\left(1-\frac{h_{3}}{k_{2}}\right)=0\;\text{under condition}\;33.\\ \begin{array}{c} {\left(T^{\left[4\right]}\right)}^{T}{\mathrm{DF}}_{r_{2}}\left(S_{4},r_{2}^{*}\right)\left(\vartheta^{\left[4\right]}\right)=\left(\frac{-a_{21}}{a_{11}},1,0\right)\left[\begin{array}{ccc} 0 & 0 & 0\\ 0 & 1 & 0\\ 0 & 0 & 0 \end{array}\right]=1\neq 0\\ {\left(T^{\left[4\right]}\right)}^{T}D^{2}{F_{r}}_{2}\left(S_{4},r_{2}^{*}\right)\left(\vartheta^{\left[4\right]},\vartheta^{\left[4\right]}\right)=\left(\frac{-a_{21}}{a_{11}},1,0\right)\left[\begin{array}{c} 0\\ \frac{-2r_{2}^{*}}{k_{2}}\\ \frac{-2c\alpha_{3}\left(1+w\right)}{{\left(1+w+ch_{3}\right)}^{3}}+\beta {\left(\frac{a_{32}}{\beta h_{3}+\gamma_{3}}\right)}^{2} \end{array}\right]=\frac{-2r_{2}^{*}}{k_{2}}\neq 0, \end{array} \end{array}$$

Therefore, there is a TB around $S_{4}$ with the parameter $r_{2}^{***}$ .

Theorem 17. The phn undergoes a Hopf bifurcation at the coexistence point $S_{5}$ to the bifurcation parameter $w^{*}$ if

$${\left[\mathrm{Det}\;]\right|}_{w=w^{*}}>0,$$ (34)

$$b\alpha_{1}p_{4}h_{4}+a\alpha_{2}p_{4}h_{4}\neq \alpha_{1}h_{4}\left(1+w^{*}+ah_{4}\right)+\alpha_{2}p_{4}\left(1+w^{*}+bp_{4}\right),$$ (35)

where the formula of $w^{*}$ is given in the proof.

Proof. The Jacobian matrix at $S_{5}$ with $w^{*}$ is given by

$$J\left(S_{5},w^{*}\right)=\left[\begin{array}{ccc} a_{11}\; & \frac{\alpha_{1}p_{4}\left(1+w^{*}+bp_{4}\right)}{{\left(1+w^{*}+\left(ah_{4}+bp_{4}\right)\right)}^{2}} & 0\\ \frac{\alpha_{2}h_{4}\left(1+w^{*}+ah_{4}\right)}{{\left(1+w^{*}+\left(ah_{4}+bp_{4}\right)\right)}^{2}} & a_{22} & 0\\ 0 & \frac{\alpha_{3}\left(1+w^{*}\right)}{{\left(1+w^{*}+ch_{4}\right)}^{2}}-\beta n_{4} & -\beta h_{4}-\gamma_{3} \end{array}\right],$$

where $a_{11}=\left(r_{1}-\gamma_{1}\right)-\frac{2r_{1}p_{4}}{k_{1}}+\frac{\alpha_{1}h_{4}\left(1+w^{*}+ah_{4}\right)}{{\left(1+w^{*}+\left(ah_{4}+bp_{4}\right)\right)}^{2}}$ , and $a_{22}=\left(r_{2}-\gamma_{2}\right)-\frac{2r_{2}h_{4}}{k_{2}}+\frac{\alpha_{2}p_{4}\left(1+w^{*}+bp_{4}\right)}{{\left(1+w^{*}+\left(ah_{4}+bp_{4}\right)\right)}^{2}}$ .

The Hopf bifurcation occurred if the following conditions are satisfied.

1.
${\left[\mathrm{Tr}]\right|}_{w=w^{*}}=0$ ,
2.
${\left[\mathrm{Det}]\right|}_{w=w^{*}}>0$ ,
3.
∂∂w[Re(λ1,2)]w=w∗≠0 (Transversality condition),

where Tr and Det are defined in the characteristic equation given by (19). Now, we set Tr=0 to find $w^{*}$ , which gives

$$\mathrm{Tr}=0⟹\left(r_{1}-\gamma_{1}\right)-\frac{2r_{1}p_{4}}{k_{1}}+\frac{\alpha_{1}h_{4}\left(1+w+ah_{4}\right)}{{\left(1+w+\left(ah_{4}+bp_{4}\right)\right)}^{2}}-\frac{2r_{2}h_{4}}{k_{2}}+\frac{\alpha_{2}p_{4}\left(1+w+bp_{4}\right)}{{\left(1+w+\left(ah_{4}+bp_{4}\right)\right)}^{2}}+\left(r_{2}-\gamma_{2}\right)=0.$$

Let $b_{1}=\left(r_{1}-\gamma_{1}\right)+\left(r_{2}-\gamma_{2}\right)-\frac{2r_{1}p_{4}}{k_{1}}-\frac{2r_{2}h_{4}}{k_{2}}$ , $b_{2}=\left(ah_{4}+bp_{4}\right)$ , $b_{3}=\alpha_{1}ah_{4}^{2}+\alpha_{2}bp_{4}^{2}$ , $b_{4}=\left(\alpha_{1}h_{4}+\alpha_{2}p_{4}\right)$ , and v=(1+w) . Then

$$\mathrm{Tr}=0⟹b_{1}+\frac{b_{4}v+b_{3}}{{\left(v+b_{2}\right)}^{2}}=0$$

Solving the above equation for v , gives

$${\left(v+b_{2}\right)}^{2}b_{1}+b_{4}v+b_{3}=0$$

Then,

$$\begin{array}{c} {\left(v+b_{2}\right)}^{2}b_{1}+b_{4}v+b_{3}=0\\ b_{1}v^{2}+\left(2b_{1}b_{2}+b_{4}\right)v+\left(b_{1}b_{2}^{2}+b_{3}\right)=0. \end{array}$$

The discriminant of the above equation is

$$\Delta ={\left(2b_{1}b_{2}+b_{4}\right)}^{2}-4b_{1}\left(b_{1}b_{2}^{2}+b_{3}\right)=b_{4}^{2}+4b_{1}\left(b_{2}b_{4}-b_{3}\right).$$

Thus,

$$v=\frac{-\left(2b_{1}b_{2}+b_{4}\right)\pm \sqrt{\Delta }}{2b_{1}},w^{*}=v-1,b_{1}\neq 0$$

That means condition (1) is satisfied at $w^{*}$ , and the characteristic equation given by (19) can be rewritten as

$$\left(-\beta h_{4}-\gamma_{3}-\lambda \right)\left[\lambda^{2}+\mathrm{Det}\right]=0$$

Solving the above equation yields $\lambda_{1}=-\beta h_{4}-\gamma_{3}$ , $\lambda_{2,3}=\pm i\sqrt{\mathrm{Det}}$ . Clearly $\lambda_{2}$ and $\lambda_{3}$ are complex conjugates if condition 34 is satisfied. In addition, the general roots of Eq. (19) in the neighborhood of $w^{*}$ as $\lambda_{2,3}=\frac{\mathrm{Tr}\pm i\sqrt{{\mathrm{Tr}}^{2}-4\mathrm{Det}}}{2}$ , then

∂∂w[Re(λ2,3)]w=w∗=bα1p4h4+aα2p4h4−α1h4(1+w∗+ah4)−α2p4(1+w∗+bp4)(1+w∗+(ah4+bp4))3≠0under condition35.

So, phn system undergoes a Hopf bifurcation at $S_{5}$ with the bifurcation parameter $w^{*}$ .

### Numerical simulations and discussion

A numerical confirmation is carried out to complete the analytical results for phn system using MATLAB. The simulations are conducted by using the following set of parameters.

$$r_{1}=0.56,k_{1}=10,\alpha_{1}=0.04,w=3,a=0.02,b=0.03\;\gamma_{1}=0.04,r_{2}=0.18,k_{2}=6,\alpha_{2}=0.08=0.4,\gamma_{2}=0.03,\alpha_{3}=0.01,\beta =0.00157,\gamma_{3}=0.0018,c=0.01.$$ (36)

**Figure 2.**
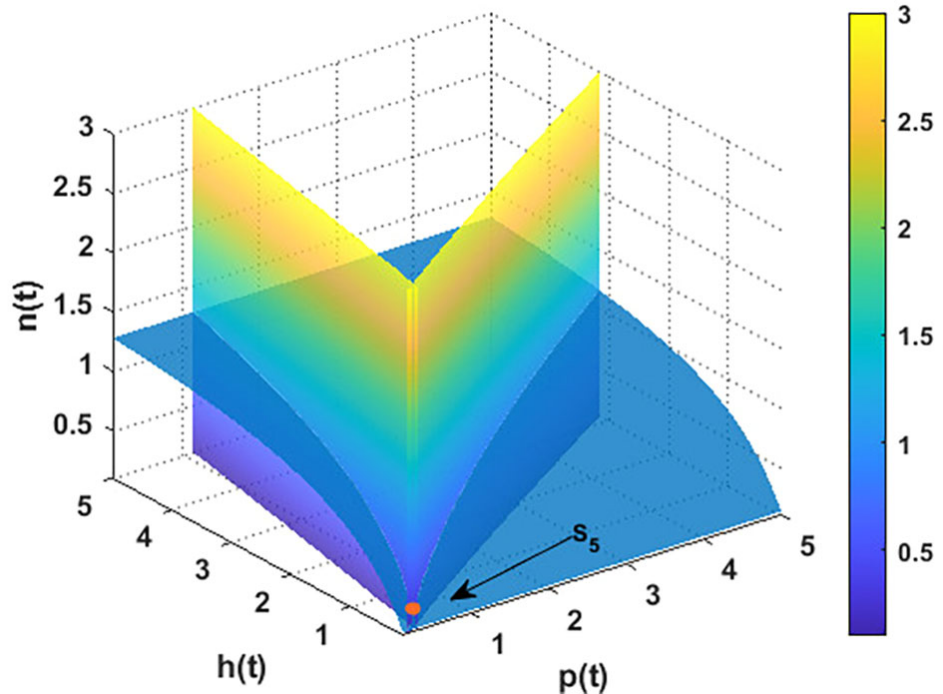
The nullclines of phn system with the parameters listed in Eq. (36).

**Figure 3.**
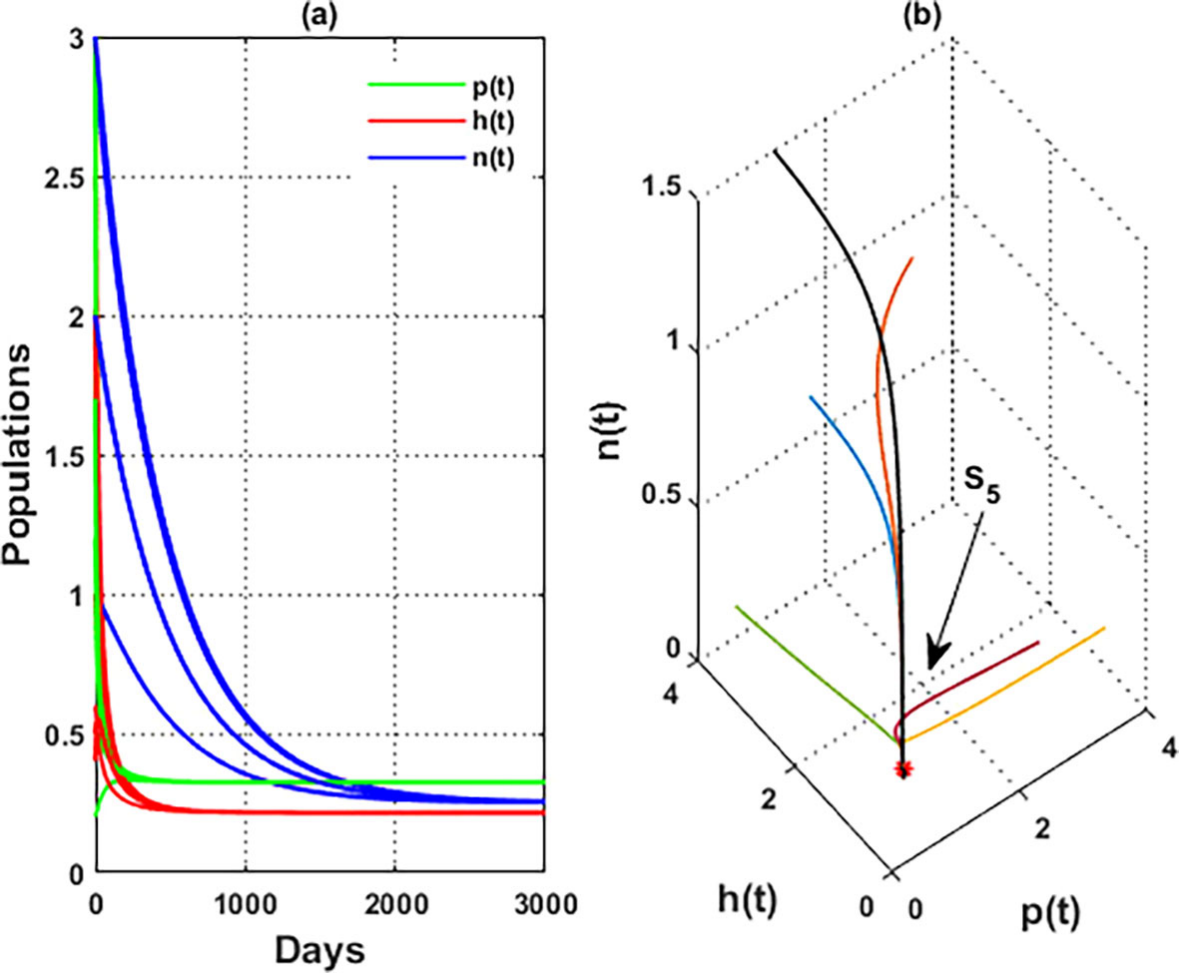
The global stability of $S_{5}=\left(0.21,0.11,0.23\right)$ .

For the parameters listed above in Eq. (36), the nullclines of phn system are indicated in Figure 2. The figure depicts the coexistence point $S_{5}=\left(p_{4},h_{4},n_{4}\right)=\left(0.21,0.11,0.23\right)$ , while Figure 3 illustrates the global behavior of $S_{5}$ . That means the parameters listed in Eq. (36) corroborate the findings of Theorem 12, which shows that all other points act as saddle points, except for $S_{5}$ . These findings also confirm the uniform persistence for phn system, which confirms the output of Theorem 3.

**Figure 4.**
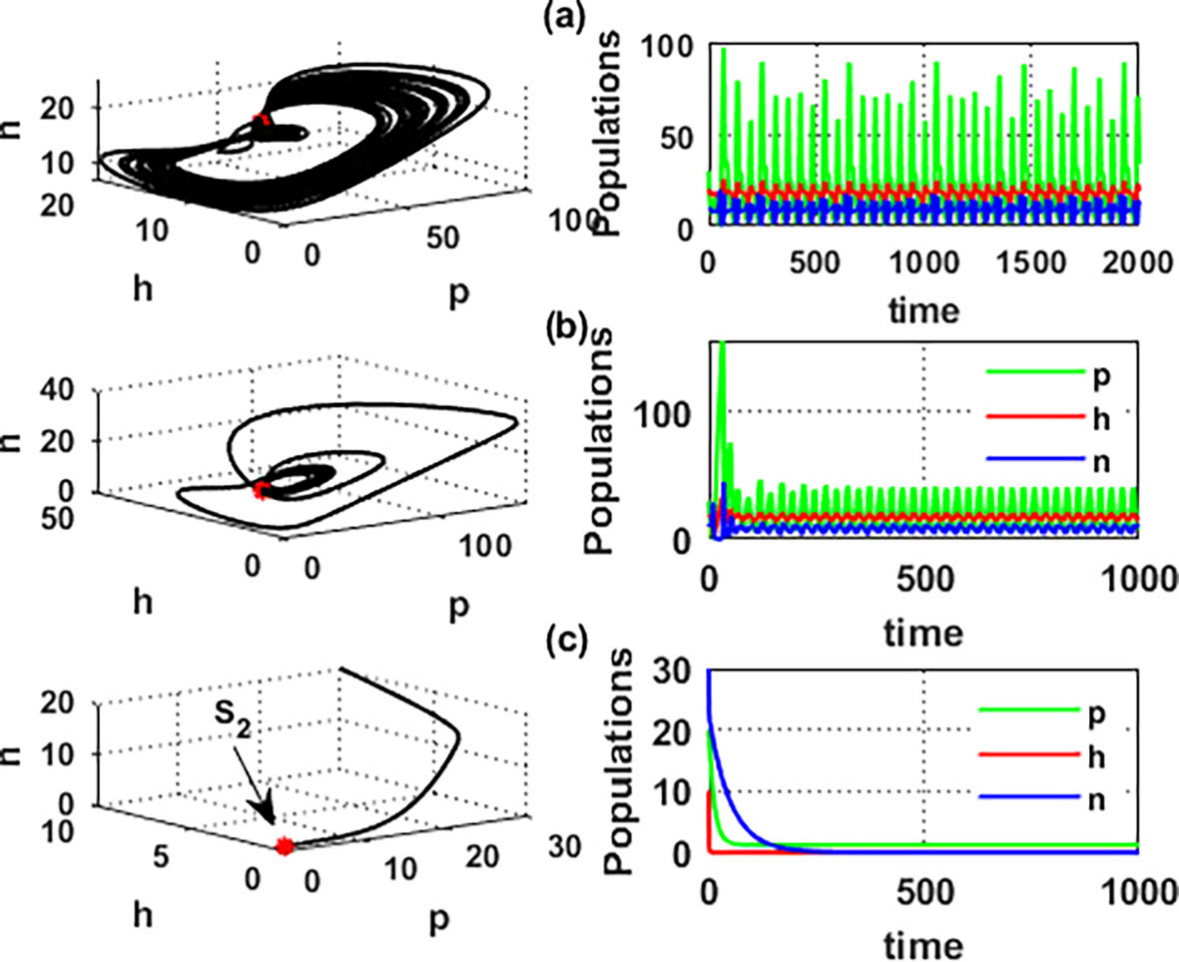
The effect of various wind speeds w .

**Figure 5.**
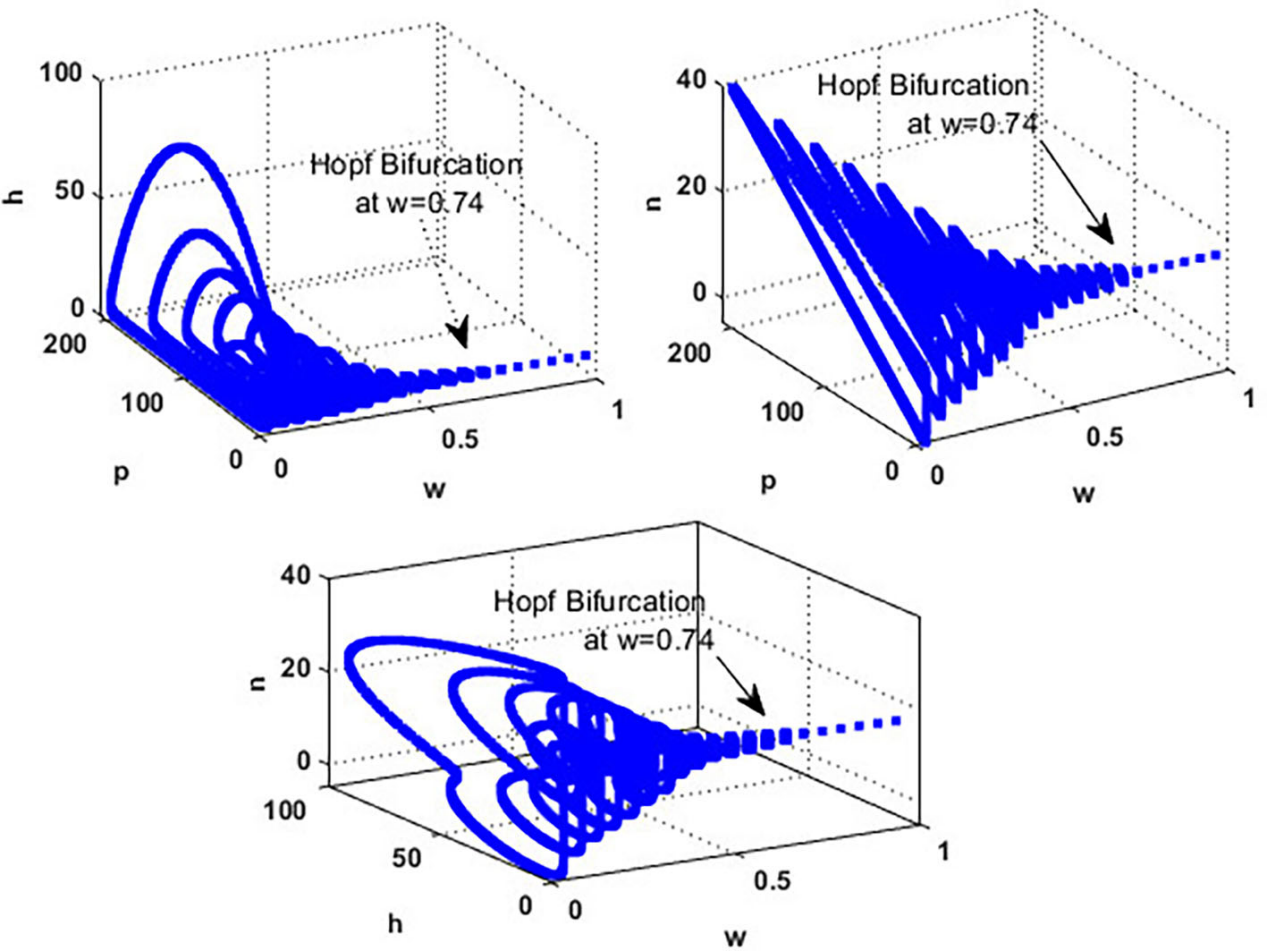
Hopf bifurcation at w=0.74 .

An important parameter to investigate is the effect of the wind level (w) on the interaction of blooming plants p(t) , honey honeybees h(t), and honey production n(t) . We address two aspects of w : first, the extent to which it influences the species densities in the inner equilibrium, and second, how it can alter phn system’s stability. For a low level of wind flow, i.e., 0<w<0.017, the solution of the phn system approaches a chaotic attractor, see Figure 4(a). While in the interval 0.017<w≤0.74 , the solution of the phn system converges to a limit cycle, see Figure 4(b) and Figure 5. Consequently, for the region 0.74<w<3.4 , the solution stabilized at the coexistence point $S_{5}$ , see Figure 2, which was plotted when w=3 . Finally, for a high level of wind speed, i.e., for w>3.4 , phn system loses two of its components, and the solution in this case settles down to the blooming plants’ point $S_{2}=\left(0.16,0,0\right)$ , see Figure 4(c). This indicates that when the wind speed is light, the wind helps pollinate or carries the seeds of flowering plants from one place to another, aiding their reproduction. Therefore, the presence of flowering plants contributes to the system’s persistence. In contrast, for a high wind flow, we see that the populations of honeybees and the production of honey face extinction.

**Figure 6.**
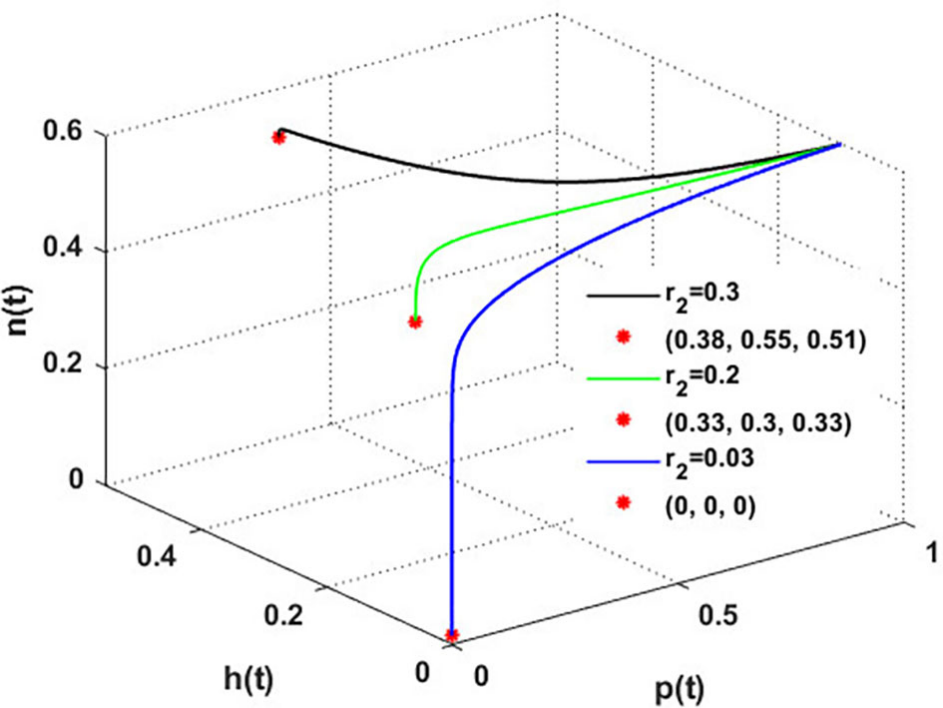
The effect of varying $r_{2}$ .

**Figure 7.**
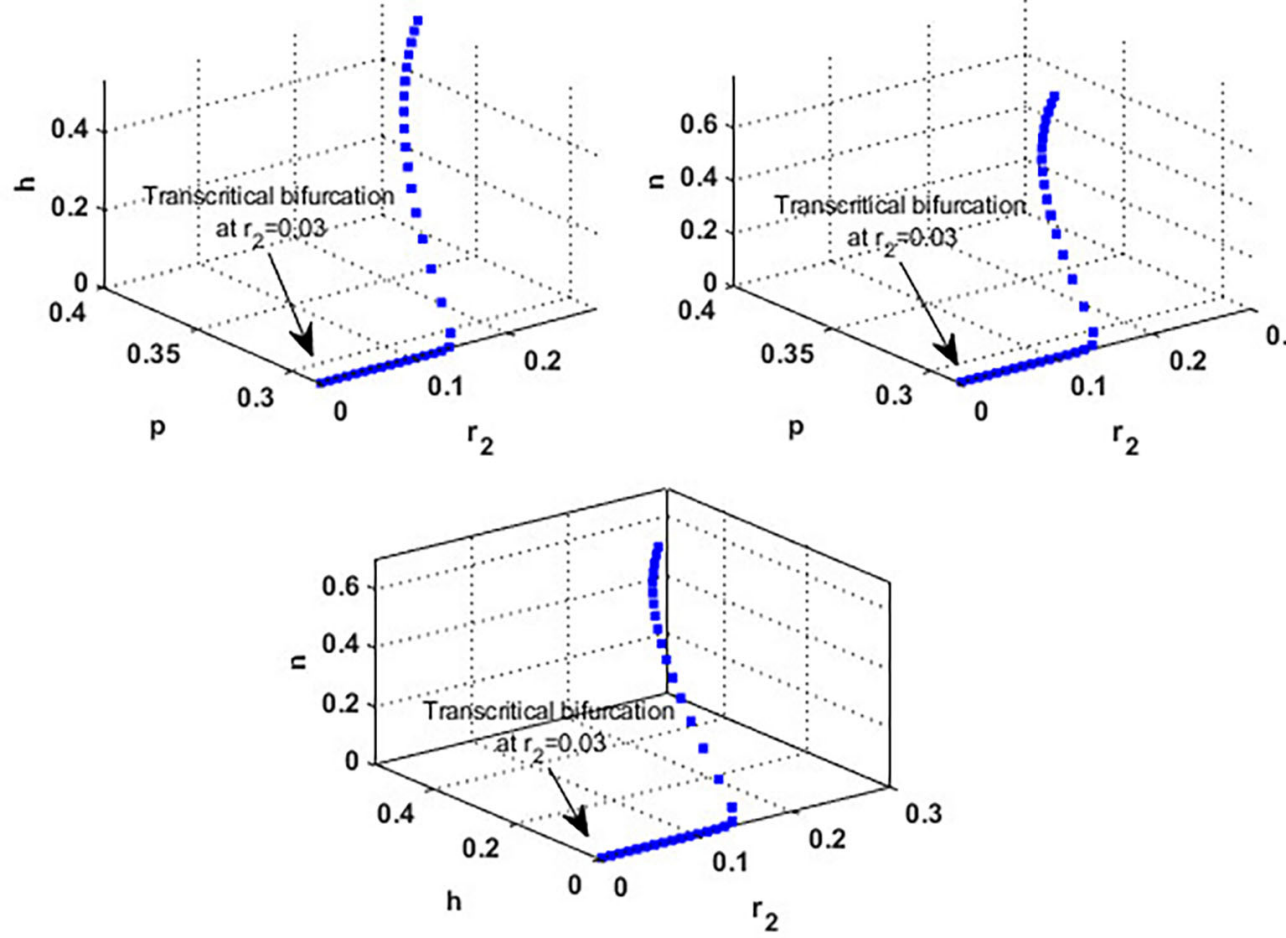
Transcritical bifurcation at $r_{2}=0.03$ .

**Figure 8.**
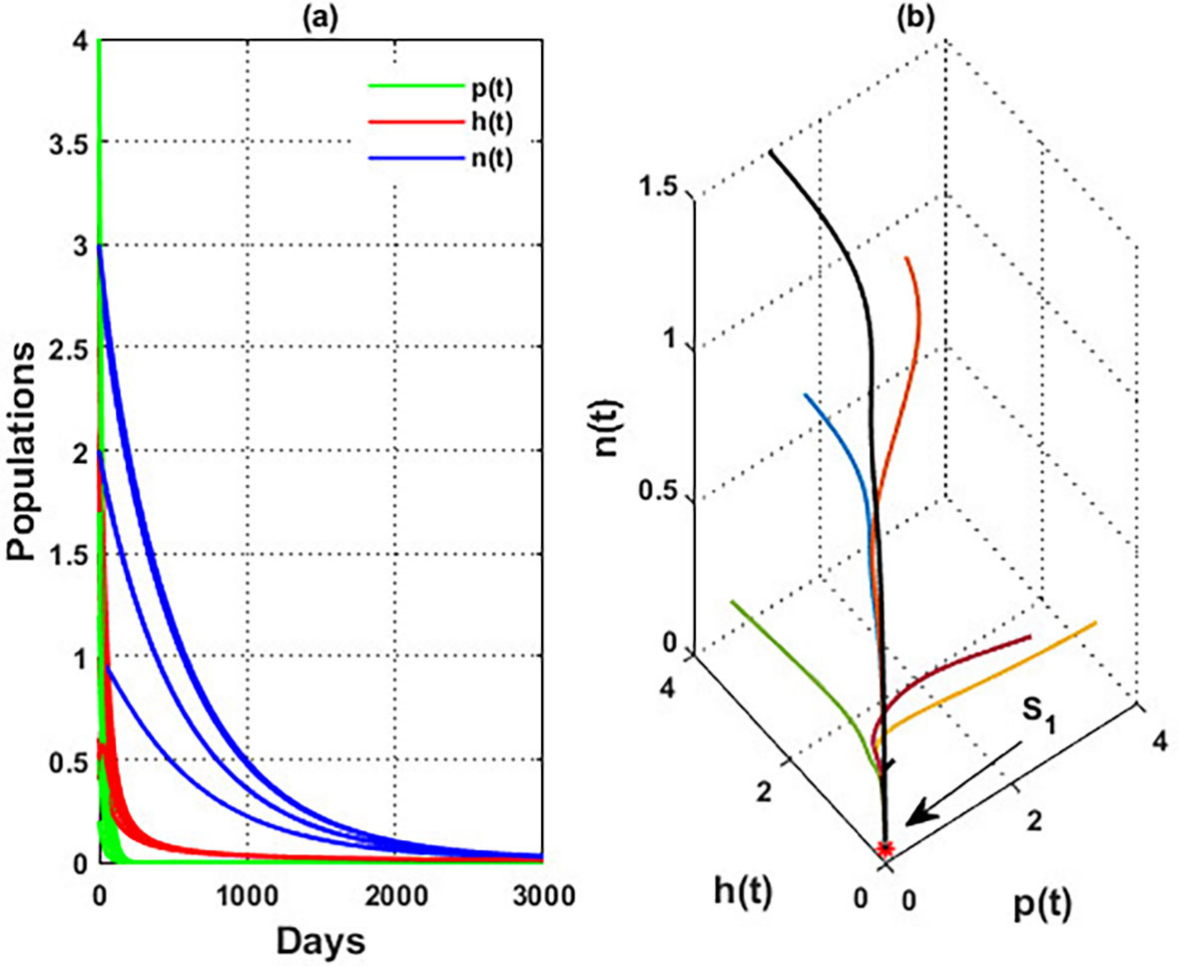
The global stability of $S_{1}$ .

Now, we have the intrinsic growth rate of the honeybee population ( $r_{2})$ , which is an essential quantity to discuss since it can potentially influence the population’s densities of blooming plants, honeybees, and honey production. It is clear from Figure 6 that the solution of phn system settles down to the coexistence point $S_{5}$ for $r_{2}>0.03$ , while it stabilized at the extinction point $S_{1}=\left(0,0,0\right)$ for $r_{2}\leq 0.03$ . This result confirms the occurrence of a transcritical bifurcation at $r_{2}=r_{2}^{\mathrm{TB}}=0.03$ , which confirms the output of Theorem 13, see Figure 7. Further, Figure 8 indicates the global behavior of the extinction point $S_{1}$ . This result confirms the global stability condition of $S_{1}$ which has honeybee stated in Theorem 8. Further, this result shows that $r_{2}$ is a critical parameter that impacts the continuity of the whole system’s coexistence.

**Figure 9.**
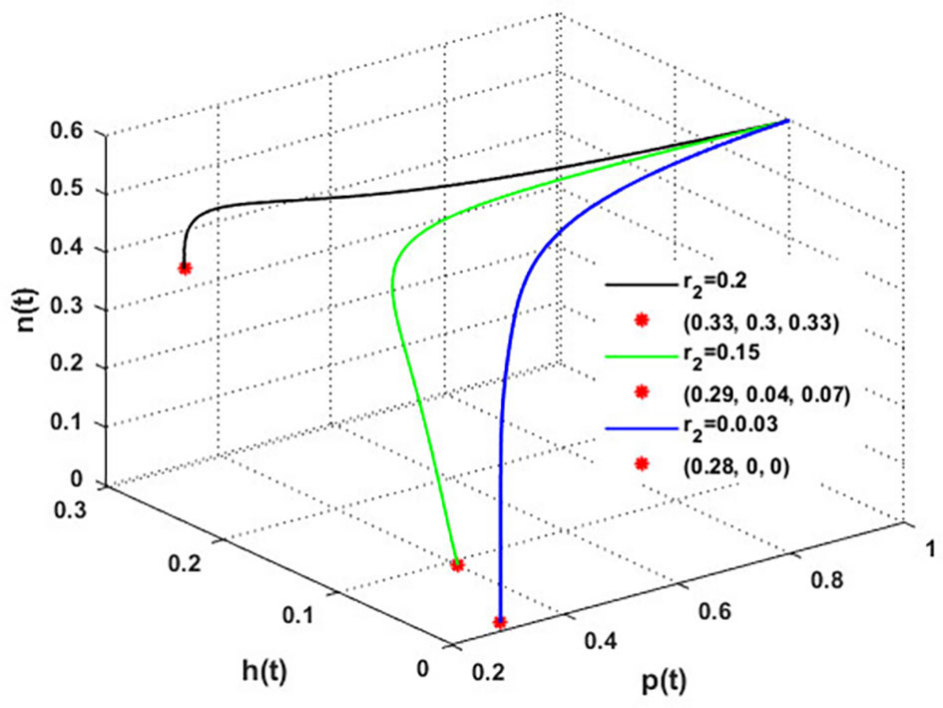
The effect of varying $r_{1}$ .

**Figure 10.**
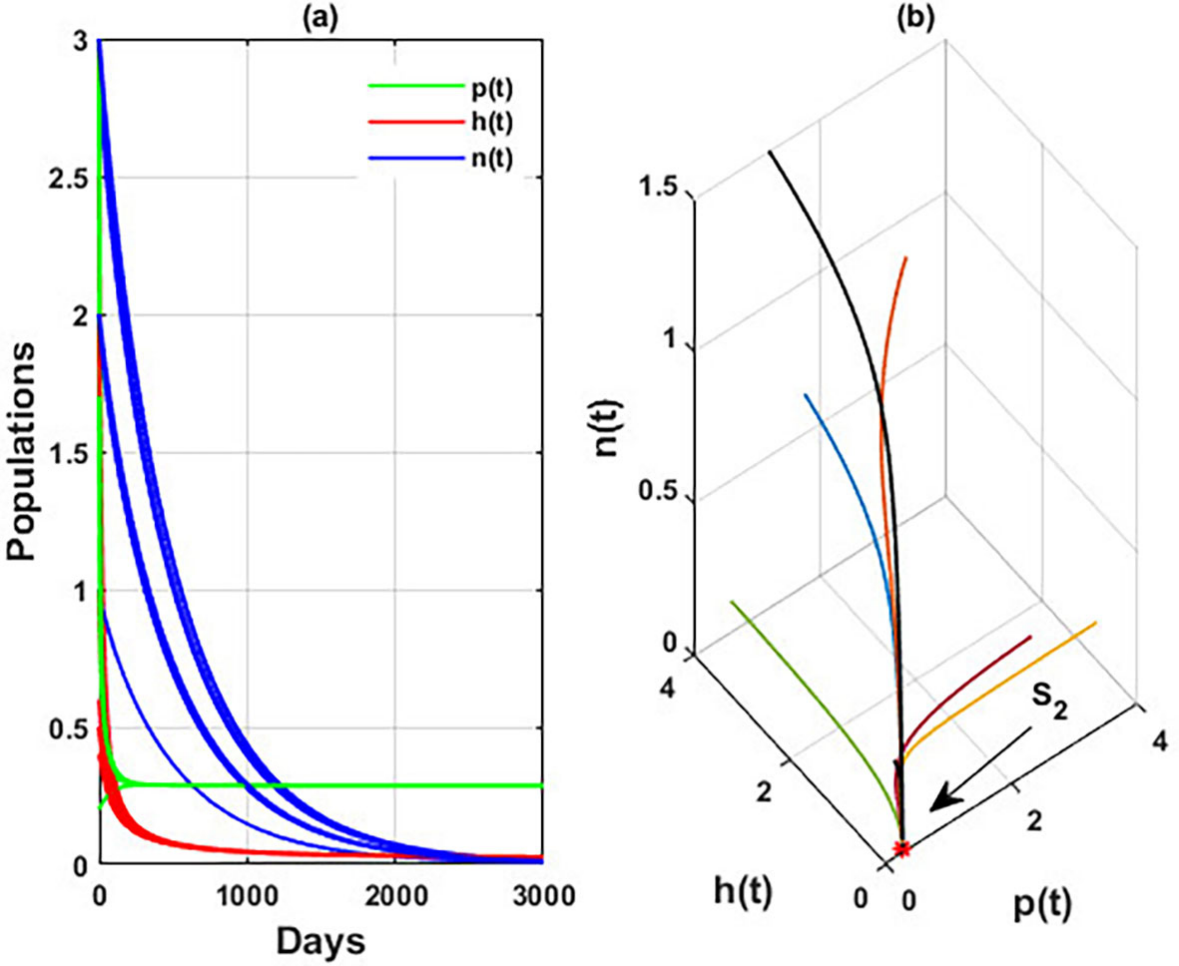
The global stability of $S_{2}$ .

The effect of varying the intrinsic growth rate $r_{1}$ of the population of blooming plants is investigated in Figure 9. The figure indicates that the solution of phn system converges to the blooming plants point $S_{2}=\left(0.28,0,0\right)$ for $r_{1}\leq 0.04$ , which means the system faces an occurrence of transcritical bifurcations at $r_{1}=r_{1}^{\mathrm{TB}}$ = 0.04, which confirms the output of Theorem 14. So, phn system loses two of its components for $r_{1}\leq 0.04$ . While for $r_{1}>0.04$ , phn system converges to the coexistence point $S_{5}$ . Further, the global stability of $S_{2}$ is illustrated in Figure 10. We can conclude that the intrinsic growth rate $r_{1}$ of the blooming plants population is a critical parameter affecting the honeybees’ persistence and honey production.

**Figure 11.**
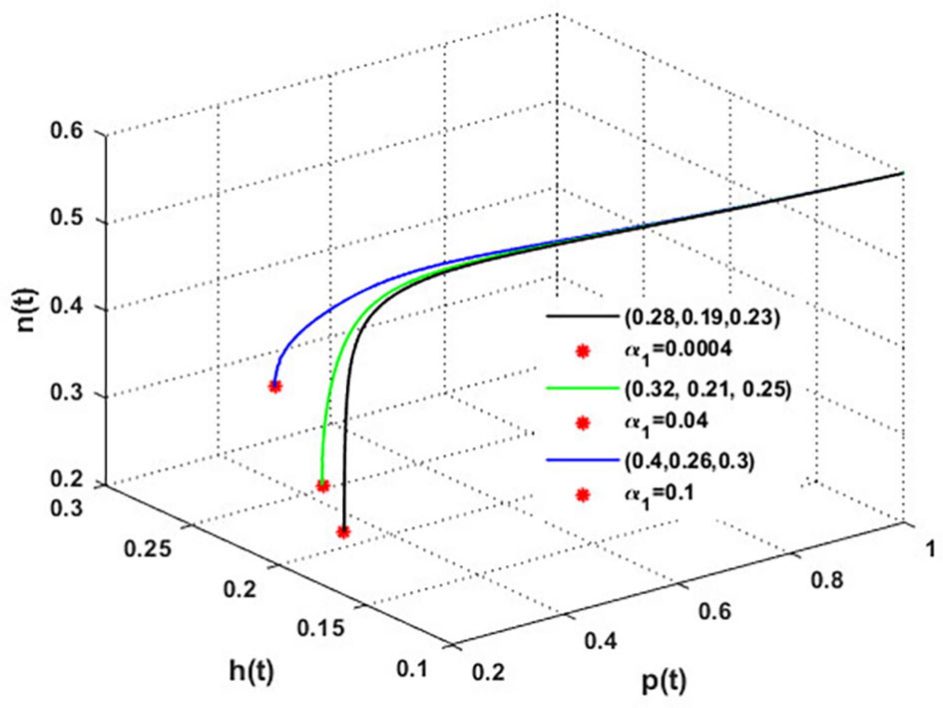
The effect of varying $\alpha_{1}$ .

**Figure 12.**
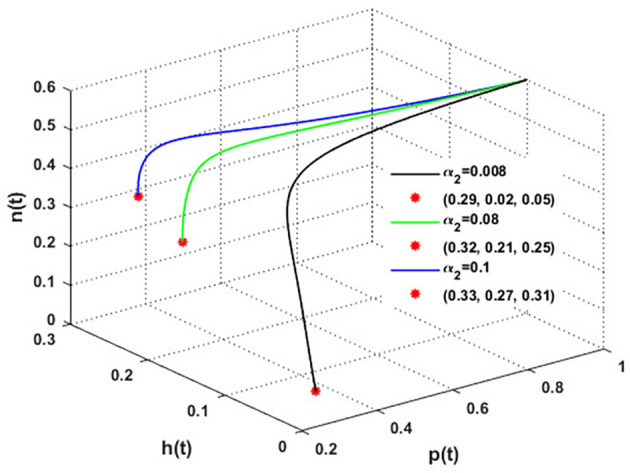
The effect of varying $\alpha_{2}$ .

The mutualistic rates between the honeybee population and the blossoming plants, $\alpha_{1}$ and $\alpha_{2}$ , are examined in Figures 11 and 12. The density of blossoming plants, the honeybee population, and honey production are all improved as a result of the increase in mutualistic rates.

**Figure 13.**
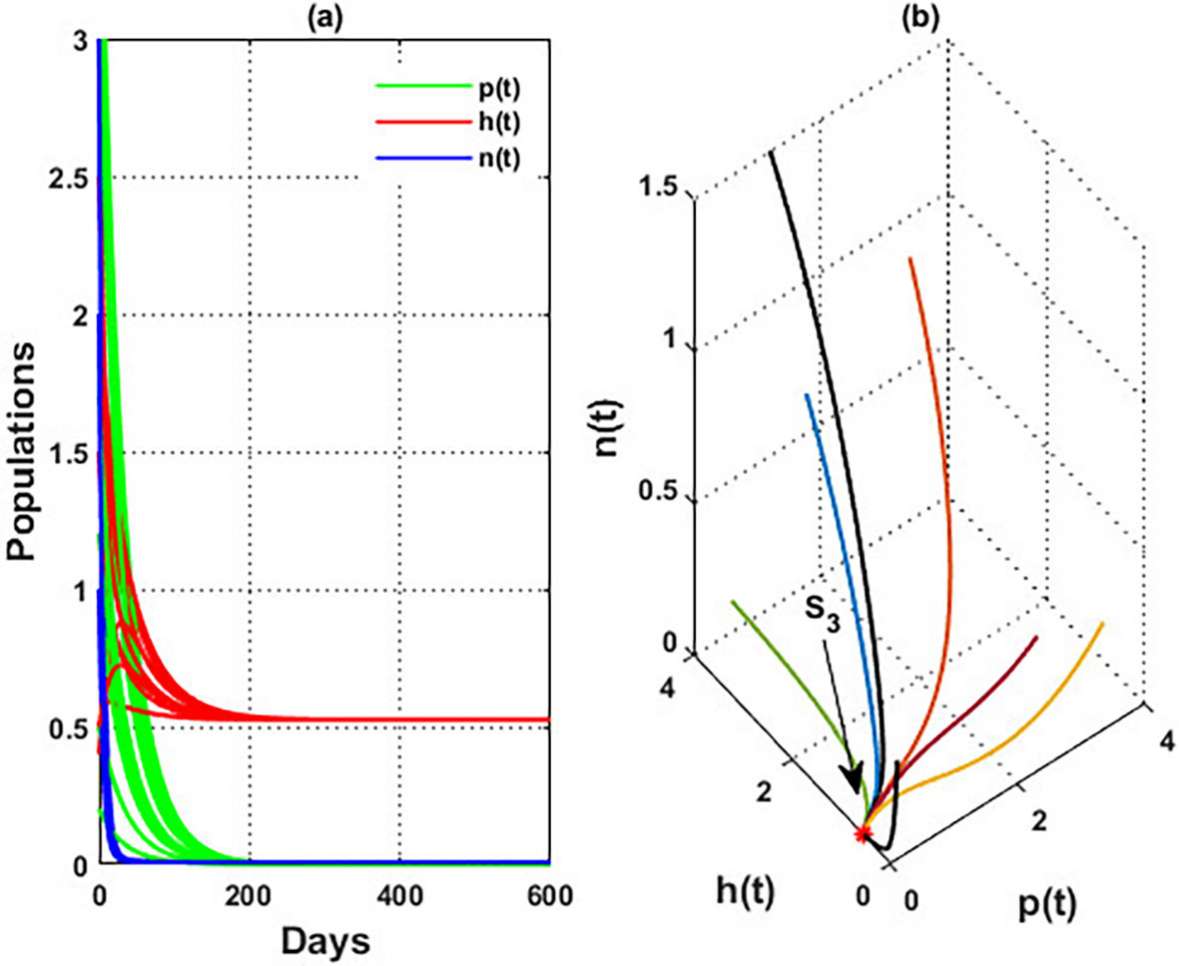
The global stability of $S_{3}$ when $\gamma_{1}=0.57$ .

A rise in the depletion rate of the population of blossoming plants population $\gamma_{1}$ (when $\gamma_{1}=0.57)$ leads to losses of the honeybee population and honey in phn system, and the solution stabilized at the honeybee point $S_{3}=\left(0,0.52,0\right)$ from various initial conditions. This result confirms the global stability theorem of $S_{3}$ which was stated in Theorem 10. As a result, the continued presence of a blossoming plant population significantly impacts the persistence of honey production since the flowering plants are the primary source of sustenance for pollinators. See Figure 13.

**Figure 14.**
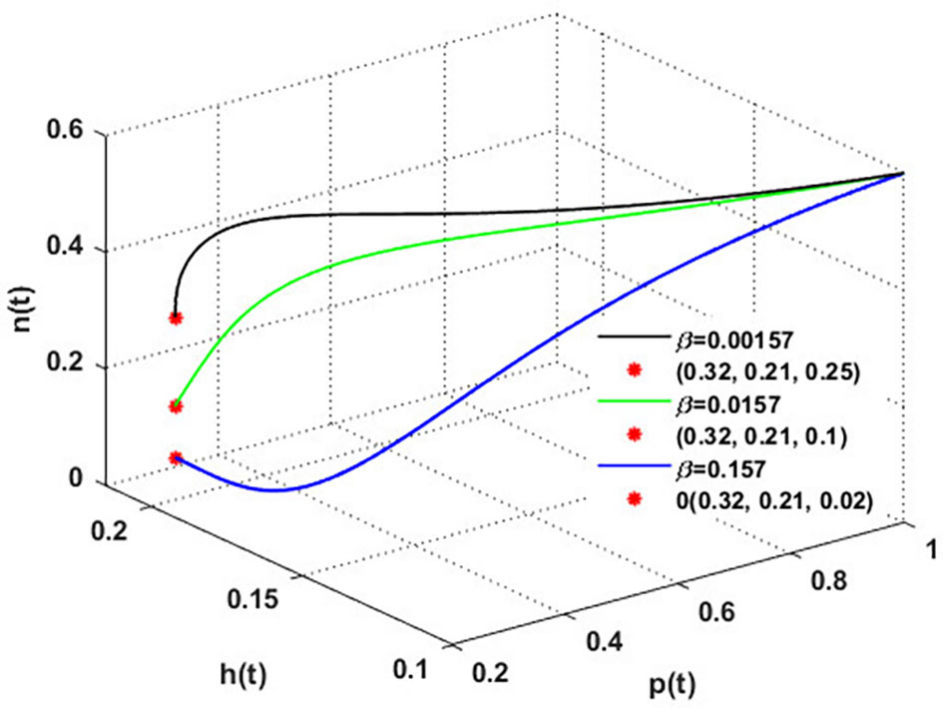
The effect of varying β .

To establish the effect of β on the dynamics of phn , Figure 14 has been drawn with three values of the rate at which honeybees consume honey to survive, i.e., β . The figure shows the solutions settling down to the coexistence point $S_{5}$ . The increase in β substantially results in a decline in honey output.

**Figure 15.**
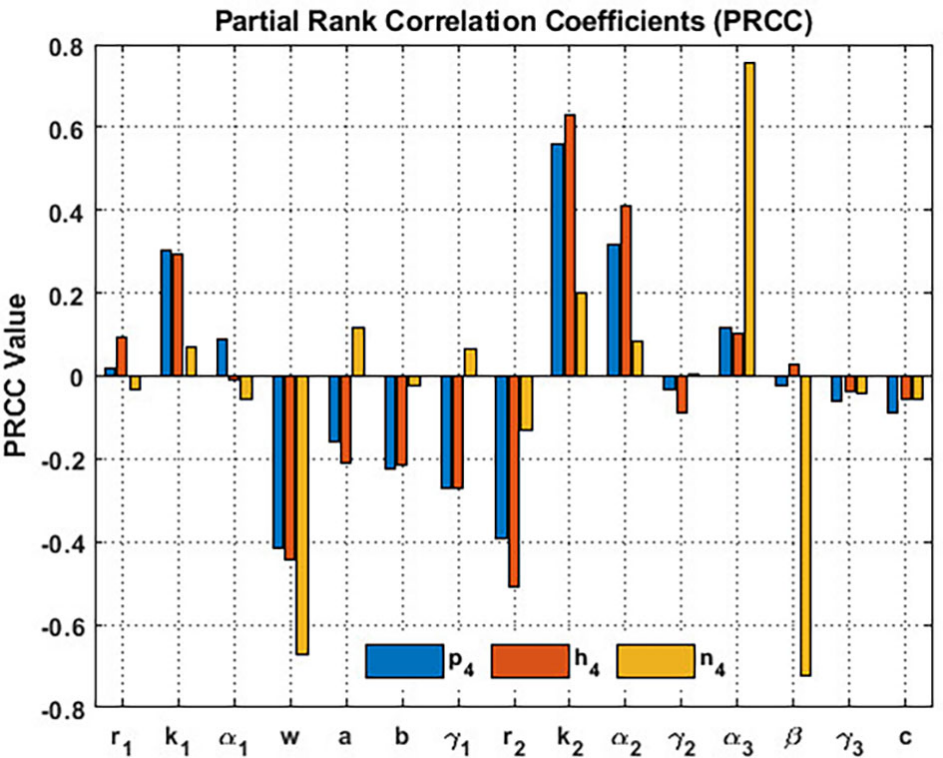
The sensitivity of the data given in Eq. 36 relative to the $S_{5}=\left(p_{4},h_{4},n_{4}\right)$ .

In order to investigate the sensitivity of the coexistence point $S_{5}=\left(p_{4},h_{4},n_{4}\right)$ of phn system, we implement partial rank correlation coefficients (PRCC). The parameters $r_{1},k_{1},\alpha_{1},w,a,b,\gamma_{1},r_{2},k_{2},\alpha_{2},\gamma_{2},\beta ,\gamma_{3},c$ and $\alpha_{3}$ serve as input parameters, whereas $p_{4},h_{4},$ and $n_{4}$ the output variables. We subsequently generate Figure 15 by utilizing the parameter set in Eq. (36). Blossoming plants and the honeybee population demonstrate heightened sensitivity to honeybees’ carrying capacity $k_{2}$ and the corresponding value of honeybee nutrients from blooming plants $\alpha_{2}$ . While the honey production determines heightened sensitivity to $\alpha_{3}$ . On the other hand, the wind flow, i.e., w, significantly influences $p_{4}$ , $h_{4}$ and $n_{4}$ . The wind flow significantly reduces the blooming plants, honeybee population, and honey production. It can be inferred that the wind flow is a critical parameter that influences the coexistence of $p_{4},h_{4},$ and $n_{4}$ , see Figure 15.

## Conclusion

Wind flow profoundly affects blossoming plants, honeybee populations, and honey production dynamics, and impacts ecosystem stability. An ODE mathematical model has been studied to understand these dynamics. The solution of phn system has been established to possess the fundamental attributes, such as positivity and persistence, boundedness, local and global stability, and bifurcation. Numerical results indicated that the honeybee species may be extinct due to increased wind velocities within specific parameter ranges. Furthermore, the coexistence equilibrium becomes unstable as a result of a Hopf bifurcation when a low wind flow induces periodic oscillations. Further, the simulations indicated that the threshold values for the transcritical bifurcation have been precisely determined at a decreased honeybee and blooming plant growth rate. However, the system may reach a point where both the blooming plant and the honeybee populations are no longer viable due to an elevated mortality rate of flowering plants. On the other hand, the increase in mutualistic rates between the honeybee population and the blooming plants has a regenerative effect, supporting the sustainability of the honeybee–honey production system.

## Data Availability

This study relies on numerical data generated from the proposed mathematical model. This data includes initial coefficients, initial conditions, and numerical simulation outputs (tables and figures). All of this data is not derived from field measurements but was generated programmatically for the purposes of theoretical analysis and numerical simulation of the system under study. This aligns with the general trend of sharing research data to enhance replication and reuse within the scientific community. All data underlying the results are available as part of the article, and no additional source data are required

## References

[1] Dean AM : A simple model of mutualism. *Am. Nat.* 1983;121(3):409–417. 10.1086/284069

[2] Ollerton J Winfree R Tarrant S : How many flowering plants are pollinated by animals? *Oikos.* 2011;120(3):321–326. 10.1111/j.1600-0706.2010.18644.x

[3] Lee Y-D Yokoi T Nakazawa T : A pollinator crisis can decrease plant abundance despite pollinators being herbivores at the larval stage. *Sci. Rep.* 2024;14(1):18523. 10.1038/s41598-024-69537-7 39122794 PMC11316071

[4] Fishman MA Hadany L : Plant–pollinator population dynamics. *Theor. Popul. Biol.* 2010;78(4):270–277. 10.1016/j.tpb.2010.08.002 20736029

[5] Jawad S Thirthar AA Nisar KS : The impact of climate change on flowering plants-bees-Vespa orientalis model. *Results Control Optim.* 2025;20:100583. 10.1016/j.rico.2025.100583

[6] Biswas A Medda R Pal S : Dynamics of predatory effect on saturated plant–pollinator mutualistic relationship. *Chaos An Interdiscip. J. Nonlinear Sci.* 2025;35(2). 10.1063/5.0233838 39899584

[7] Hakeem E Jawad S Ali AH : How mathematical models might predict desertification from global warming and dust pollutants. *MethodsX.* 2025;14:103259. 10.1016/j.mex.2025.103259 40165851 PMC11957603

[8] Lever JJ Nes EH van Scheffer M : The sudden collapse of pollinator communities. *Ecol. Lett.* 2014;17(3):350–359. 10.1111/ele.12236 24386999

[9] Al Nuaimi M Jawad S : Modelling and stability analysis of the competitional ecological model with harvesting. *Commun. Math. Biol. Neurosci.* 2022;2022:Article-ID.

[10] Shalan RN Shireen R Lafta AH : Discrete an SIS model with immigrants and treatment. *J. Interdiscip. Math.* 2020;24:1201–1206. 10.1080/09720502.2020.1814496

[11] Ali A Jawad S : Stability analysis of the depletion of dissolved oxygen for the Phytoplankton-Zooplankton model in an aquatic environment. *Iraqi J. Sci.* 2024;2736–2748. 10.24996/ijs.2024.65.5.31

[12] Nezar F Jawad S Winter M : Stability analysis of excessive carbon dioxide gas emission model through following reforestation policy in low-density forest biomass. *Baghdad Sci. J.* 2025;22(4):1335–1353.

[13] Cresswell JE : A demographic approach to evaluating the impact of stressors on bumble bee colonies. *Ecol. Entomol.* 2017;42(2):221–229. 10.1111/een.12376

[14] Thirthar AA Panja P Abdeljawad T : Impact of alarm signals and mutualistic interactions in a food chain model of oxpeckers, zebras, and lions. *Partial Differ. Equations Appl. Math.* 2025;14:101189. 10.1016/j.padiff.2025.101189

[15] Jawad S Roy S Thirthar AA : Deterministic and stochastic risks assessment of excessive CO2 emission on forest biomass under weak allee effect. *J. Appl. Math. Comput.* 2025;71:9129–9156. 10.1007/s12190-025-02640-8

[16] Thirthar AA Alaoui AL Roy S : Fractional and stochastic dynamics of predator–prey systems: The role of fear and global warming. *Eur. Phys. J. B.* 2025;98(7):1–21. 10.1140/epjb/s10051-025-00992-5

[17] Ahmed M Jawad S Das D : Impact of dust storms on plant biomass: Model structure and dynamic study. *Alex. Eng. J.* 2025;126:605–622. 10.1016/j.aej.2025.04.058

[18] Balfour NJ Ratnieks FLW : Wind Alters Plant-Pollinator Community Structure, Bee Foraging Rate & Movements Between Plants. *Behav. Ecol.* 2025;36:araf067. 10.1093/beheco/araf067 40666820 PMC12260157

[19] Friedman J Barrett SCH : Wind of change: new insights on the ecology and evolution of pollination and mating in wind-pollinated plants. *Ann. Bot.* 2009;103(9):1515–1527. 10.1093/aob/mcp035 19218583 PMC2701749

[20] Chen F : On a nonlinear nonautonomous predator–prey model with diffusion and distributed delay. *J. Comput. Appl. Math.* 2005;180(1):33–49. 10.1016/j.cam.2004.10.001

[21] Place CM : *Dynamical Systems: Differential Equations, Maps, and Chaotic Behaviour.* Routledge;2017.

[22] Aamer Z Jawad S Batiha B : Evaluation of the Dynamics of Psychological Panic Factor, Glucose Risk and Estrogen Effects on Breast Cancer Model. *Computation.* 2024;12(8):160. 10.3390/computation12080160

[23] Ahmed M Jawad S : Bifurcation analysis of the role of good and bad bacteria in the decomposing toxins in the intestine with the impact of antibiotic and probiotics supplement. *AIP Conference Proceedings.* AIP Publishing;2024.

[24] Thirthara AA Jawadb S Shahc K : How does media coverage affect a COVID-19 pandemic model with direct and indirect transmission? 2024.

[25] Javaid Y Jawad S Ahmed R : Dynamic complexity of a discretized predator-prey system with Allee effect and herd behaviour. *Appl. Math. Sci. Eng.* 2024;32(1):2420953. 10.1080/27690911.2024.2420953

[26] Roy S AL-Jaf DS Thirthar AA : The impact of human shields in autonomous and non-autonomous prey-predator models with modified Cosner functional response. *Math. Comput. Simul.* 2025;242:54–73. 10.1016/j.matcom.2025.11.016

