# Supplementary material for: Impact of high wind speed on blooming plants-honeybees-honey production model 
Source: F1000Res. 2026 Apr 29;14:1459. Originally published 2025 Dec 26. [Version 2] doi: 10.12688/f1000research.172134.2 (PMC13173679; doi:10.12688/f1000research.172134.2)
Supplement: Peer review history

# Reviewer report: Reviewer response for version 2

**DOI:** 10.5256/f1000research.198543.r480277

**Published:** 2026-05-12

**Authors:** Vinoth Seralan

**Affiliations:** 1 SRM Institute of Science and Technology, Chennai, Tamil Nadu, India

I appreciate the authors for carried out all my comments and suggestions.

Is the work clearly and accurately presented and does it cite the current literature?

Yes

If applicable, is the statistical analysis and its interpretation appropriate?

No

Are all the source data underlying the results available to ensure full reproducibility?

No

Is the study design appropriate and is the work technically sound?

Yes

Are the conclusions drawn adequately supported by the results?

Yes

Are sufficient details of methods and analysis provided to allow replication by others?

Yes

Reviewer Expertise:

Mathematical biology, chaos theory

I confirm that I have read this submission and believe that I have an appropriate level of expertise to confirm that it is of an acceptable scientific standard.

# Reviewer report: Reviewer response for version 1

**DOI:** 10.5256/f1000research.189832.r464608

**Published:** 2026-03-25

**Authors:** Vinoth Seralan

**Affiliations:** 1 SRM Institute of Science and Technology, Chennai, Tamil Nadu, India

In this work, the authors studied the dynamics of interactions between blooming plants and honeybee populations, which is crucial for addressing honeybee decline and sustainable ecosystems. The mathematical model on dynamics of a blooming plant, honeybee population, and honey production symbiosis, which is special emphasis on the effect of high-speed wind flow were investigated. Furthermore, the existence of two difference bifurcation phenomena such as the transcritical and Hopf bifurcations have been discussed.   I have few comments as follows:

1. In abstract, the authors mentioned, Habitat loss, diseases, climate change, pesticides, and predation have all contributed to the decline of pollinator species. Although, it has been discussed in the manuscript. More discussions to be added.
2. There are some typos, please correct it. For example. blooming plants p, honey honeybees h (honey is repeated). The biological meaning of the parameter w is not mentioned.
3. The derivation of theoretical bifurcation condition both bifurcations are correct but add a proof write more lines that are numerically satisfied.
4. In phase portrait Figure 4 (a), it has been shown as chaotic trajectories. But in bifurcation diagram Figure 5, its look like a periodic attractor.
5. The numerical simulation section provides strong supporting evidence whenever needed, which is a positive aspect. The prescribed-time results need more biologically justified.
6. The authors suggested to compare their finding by referring some related papers about implementing control of habitat loss in a separate remark. Discuss about sustainable production of honey.
7. Please summary the conclusions with more biological justification and point out how different the model is from other existing models.

Is the work clearly and accurately presented and does it cite the current literature?

Yes

If applicable, is the statistical analysis and its interpretation appropriate?

No

Are all the source data underlying the results available to ensure full reproducibility?

No

Is the study design appropriate and is the work technically sound?

Yes

Are the conclusions drawn adequately supported by the results?

Yes

Are sufficient details of methods and analysis provided to allow replication by others?

Yes

Reviewer Expertise:

Mathematical biology, chaos theory

I confirm that I have read this submission and believe that I have an appropriate level of expertise to confirm that it is of an acceptable scientific standard, however I have significant reservations, as outlined above.

# Response

**Published:** 2026-04-06

**Authors:** Shireen Jawad

**Affiliations:** Mathematics, University of Baghdad, Baghdad, Baghdad Governorate, Iraq

Thank you very much for giving us the opportunity to revise our manuscript. We really appreciate the helpful comments from the learned reviewers, which helped improve our manuscript. We have revised the manuscript in response to each comment and provided our responses below. We sincerely hope the revised manuscript meets your expectations, and we look forward to hearing from you at your convenience.

- In abstract, the authors mentioned, Habitat loss, diseases, climate change, pesticides, and predation have all contributed to the decline of pollinator species. Although, it has been discussed in the manuscript. More discussions to be added.

Response: Thank you for your comment. More discussion of all contributions to the decline of pollinator species has been added. Please see the introduction section. We consider the impact of pesticides in our next study; we mention this at the end of the conclusion section.

- There are some typos, please correct it. For example. blooming plants p, honey honeybees h (honey is repeated). The biological meaning of the parameter w is not mentioned.

Response: Thank you very much for your comment. The repeated word ‘honey’ has been removed. The biological meaning of w is clearly presented in numbers 1-2 of the “Assumptions of the model” section.

- The derivation of theoretical bifurcation condition both bifurcations are correct but add a proof write more lines that are numerically satisfied.

Response: Thank you for your valuable comment. We are glad that the reviewer finds the derivation of the theoretical bifurcation conditions to be correct. In response to your suggestion, we have expanded the numerical simulation section by showing at which specific region the bifurcations could happen.

- In phase portrait Figure 4 (a), it has been shown as chaotic trajectories. But in bifurcation diagram Figure 5, its look like a periodic attractor.

Response: Thank you for your comment. Figure 5 confirms only the accuracy of the Hopf bifurcation for case 0.017

# Response

**Published:** 2026-05-06

**Authors:** vinoth seralan

I appreciate the authors for carried out all my comments and suggestions.

# Reviewer report: Reviewer response for version 1

**DOI:** 10.5256/f1000research.189832.r464611

**Published:** 2026-03-13

**Authors:** Muhammad Sajjad Shabbir

**Affiliations:** 1 University of Engineering and Technology, Lahore, Pakistan

The article develops a mathematical model describing the interaction between blooming plants, honeybees, and honey production while considering wind speed as an environmental disturbance. The model is formulated using nonlinear ordinary differential equations with a Beddington–DeAngelis functional response. The authors analyze the system’s dynamics through theoretical methods and numerical simulations, examining properties such as stability and persistence. Bifurcation analysis is also performed to study how parameter changes affect system stability. The results indicate that high wind speeds can destabilize the ecosystem and may lead to the extinction of honeybee populations and reduced honey production.

 Overall, the manuscript is well organized, mathematically rigorous, and addresses a significant problem in mathematical ecology. I recommend it for indexing provided the following comments are addressed:

1. Parameter estimation issues: Many of the parameter values used in the simulations appear to be selected for illustrative purposes rather than derived from measured ecological data, which may affect the realism of the simulation results.
2. Limited environmental factors: The model only considers wind speed as an environmental disturbance, while other important ecological factors such as temperature variation, pesticide exposure, diseases, and habitat loss are not included.
3. Simplified ecological assumptions: Some biological processes involved in plant–pollinator interactions are simplified in the model, which may overlook complex ecological behaviors occurring in real ecosystems.
4. High mathematical complexity: Several parts of the analysis are mathematically intensive and may be difficult for readers from ecological or interdisciplinary backgrounds to fully interpret.

Is the work clearly and accurately presented and does it cite the current literature?

Yes

If applicable, is the statistical analysis and its interpretation appropriate?

Not applicable

Are all the source data underlying the results available to ensure full reproducibility?

No source data required

Is the study design appropriate and is the work technically sound?

Yes

Are the conclusions drawn adequately supported by the results?

Yes

Are sufficient details of methods and analysis provided to allow replication by others?

Yes

Reviewer Expertise:

Mathematical biology, Optimal control of epidemiological models, numerical solution of ode

I confirm that I have read this submission and believe that I have an appropriate level of expertise to confirm that it is of an acceptable scientific standard.
